# Supplementary material for: Missed opportunity for family planning counselling along the continuum of care in Arusha region, Tanzania
Source: PLoS One. 2021 Jul 14;16(7):e0250988. doi: 10.1371/journal.pone.0250988 (PMC8279341; doi:10.1371/journal.pone.0250988)
Supplement: Attachment — Submitted filename: PONE-D-20-36870_R1 R2 FP MB.pdf [file pone.0250988.s003.pdf]

PLOS ONE

Missed opportunity for family planning counselling along the continuum of care in Arusha region, Tanzania

--Manuscript Draft--

**Manuscript Number:**	PONE-D-20-36870R1
**Article Type:**	Research Article
**Full Title:**	Missed opportunity for family planning counselling along the continuum of care in Arusha region, Tanzania
**Short Title:**	Missed opportunity for family planning counselling
**Corresponding Author:**	Caroline Amour<br>KCMU College: Kilimanjaro Christian Medical University College<br>Moshi, TANZANIA, UNITED REPUBLIC OF
**Keywords:**	Missed opportunity,<br>family planning,<br>counselling,<br>Tanzania
**Abstract:**	Introduction<br><br>Introduction<br>Adequate sexual and reproductive health information is vital to women of reproductive age (WRA) 15 to 49 years, for making informed choices on their reproductive health including family planning (FP). However, many women who interact with the health system continue to miss out this vital service. The study aimed to identify the extent of provision of FP counselling at service delivery points and associated behavioral factors among women of reproductive age in two districts of Arusha region. It also determined the association between receipt of FP counselling and contraceptive usage.<br>Methods<br>Data were drawn from a cross-sectional survey of 5,208 WRA residing in two districts of Arusha region in Tanzania; conducted between January and May 2018. Multistage sampling technique was employed to select the WRA for the face-to-face interviews. FP counseling was defined as receipt of FP information by a woman during any visit at the health facility for antenatal care (ANC), or for post-natal care (PNC). Analyses on receipt of FP counselling were done on 3,116 WRA, aged 16-44 years who were in contact with health facilities in the past two years. A modified Poisson regression model was used to determine the Prevalence Ratio (PR) as a measure of association between receipt of any FP counseling and current use of modern contraception, controlling for potential confounders.<br>Results<br>Among the women that visited the health facility for any health-related visit in the past two years, 1,256 (40%) reported that they received FP counselling. Among the women who had had births in the last 30 months; 1,389 and 1,409 women had contact with the service delivery points for ANC and PNC visits respectively. Of these 31% and 26% had a missed FP counseling at ANC and PNC visit respectively. Women who were not formally employed were more likely to receive FP counselling during facility visit than others. WRA who received any FP counseling at PNC were significantly more likely to report current use of modern contraception than those who did not (adjusted PR [adj. PR] =1.28; 95% Confidence Interval [CI]: 1.09, 1.49).<br>Conclusion<br>Overall, only 40% women reported that they received any form of FP counseling when they interfaced with the healthcare system in the past two years. Informally employed women were more likely to receive FP counselling, and women who received FP counselling during PNC visits were significantly more likely to use contraceptive in comparison to the women who did not receive FP counselling. This presents a missed opportunity for prevention of unintended pregnancies and suggests a need for further integration of FP counseling into the ANC and PNC visits.
**Order of Authors:**	Caroline Amour
	Rachel N Manongi

Powered by Editorial Manager® and ProduXion Manager® from Aries Systems Corporation

	Michael J Mahande
	Bilikisu Elewonibi
	Amina Farah
	Sia Emmanuel Msuya
	Iqbal Shah
**Response to Reviewers:**	Point by point response to reviewer’s comments<br>Manuscript ID: Ms. [PONE-D-20-36870]<br>Missed opportunity for family planning counselling along the continuum of care in Arusha region, Tanzania<br><br>Journal: PLOS ONE<br><br>Subject: Author's response to reviewer’s comments<br>Dear Editor,<br>Thanks very much for the invitation to resubmit our manuscript entitled: “Missed opportunity for family planning counselling along the continuum of care in Arusha region, Tanzania”. We appreciate the reviewers’ useful comments and suggestions to improve our manuscript. We were able to address all the comments and suggestions, and therefore we are expecting that the paper can now be accepted in your journal. Please find our responses as are indicated in a red color. All comments requested have been incorporated in the main text.<br>Regarding Financial disclosures; None of the research costs or authors' salaries were funded, in whole or in part, by a tobacco company. The identity of the donor is irrelevant to editors or reviewers’ assessment of the validity of the work. The authors are not aware of any competing interests.<br><br>Caroline Amour<br>Corresponding author<br><br><br><br>Journal Requirements:<br>When submitting your revision, we need you to address these additional requirements.<br>1. Please ensure that your manuscript meets PLOS ONE's style requirements, including those for file naming. The PLOS ONE style templates can be found at https://journals.plos.org/plosone/s/file?id=wjVg/PLOSOne_formatting_sample_main_body.pdf and https://journals.plos.org/plosone/s/file?id=ba62/PLOSOne_formatting_sample_title_authors_affiliations.pdf<br>Thanks for this important observation. We have now made the necessary changes in the revised manuscript.<br>2. Thank you for stating in your financial disclosure:<br>"This project was made possible by a grant from an anonymous donor to Harvard T.H. Chan School of Public Health. The funder had no role in study design, data collection and analysis, decision to publish, or preparation of the manuscript."<br>PLOS ONE requires you to include in your manuscript further information about the funder so that any relevant competing interests can be assessed. Please respond to the following questions:<br>1.Please state whether any of the research costs or authors' salaries were funded, in whole or in part, by a tobacco company (our policy on tobacco funding is at http://journals.plos.org/plosone/s/disclosure-of-funding-sources)<br>2.Please state whether the donor has any competing interests in relation to this work (see http://journals.plos.org/plosone/s/competing-interests) .<br>3.Please state whether the identity of the donor might be considered relevant to editors or reviewers’ assessment of the validity of the work.<br>4.If the donors have no perceived or actual competing interests, please state: “The authors are not aware of any competing interests”.<br>This information should be included in your cover letter. We will amend your financial disclosure and competing interests on your behalf.<br>Thank you for the comment. None of the research costs or authors' salaries were funded, in whole or in part, by a tobacco company. The identity of the donor is

irrelevant to editors or reviewers' assessment of the validity of the work. The authors are not aware of any competing interests. This additional information has been updated in the cover letter and the revised manuscript.

3. Please provide additional details regarding participant consent. In the ethics statement in the Methods and online submission information, please ensure that you have specified what type you obtained (for instance, written or verbal, and if verbal, how it was documented and witnessed). If your study included minors, state whether you obtained consent from parents or guardians. If the need for consent was waived by the ethics committee, please include this information.
Thank you for this important observation. A written participant consent was obtained before participation in the study for women aged 18 years and older. Participants who were aged 16 – 17 an assent was requested. Consent for them to participate was sought from partners for those who were married/ cohabiting, and from parents/ guardians for those who were under parental care. This information has been added to the methods section of the revised manuscript.
4. Please consider whether participants younger than 18 years old should be referred to as 'girls' rather than 'women'.
We thank the editor for this important observation. We have now addressed this in the revised manuscript.
5. We noticed you have some minor occurrence of overlapping text with the following previous publication(s), which needs to be addressed:
- Nabirye, J., Matovu, J.K.B., Bwanika, J.B. et al. Missed opportunities for family planning counselling among HIV-positive women receiving HIV Care in Uganda. BMC Women's Health 20, 91 (2020). https://doi.org/10.1186/s12905-020-00942-6
In your revision ensure you cite all your sources (including your own works), and quote or rephrase any duplicated text outside the methods section. Further consideration is dependent on these concerns being addressed.
The authors thank the editor for this observation. We have now made the necessary citations in the revised manuscript.

Comments to the Author
1. Is the manuscript technically sound, and do the data support the conclusions?
The manuscript must describe a technically sound piece of scientific research with data that supports the conclusions. Experiments must have been conducted rigorously, with appropriate controls, replication, and sample sizes. The conclusions must be drawn appropriately based on the data presented.
Reviewer #1: Partly
Reviewer #2: Yes

____

2. Has the statistical analysis been performed appropriately and rigorously?
Reviewer #1: Yes
Reviewer #2: Yes

____

3. Have the authors made all data underlying the findings in their manuscript fully available?

The PLOS Data policy requires authors to make all data underlying the findings described in their manuscript fully available without restriction, with rare exception (please refer to the Data Availability Statement in the manuscript PDF file). The data should be provided as part of the manuscript or its supporting information, or deposited to a public repository. For example, in addition to summary statistics, the data points behind means, medians and variance measures should be available. If there are restrictions on publicly sharing data—e.g. participant privacy or use of data from a third party—those must be specified.
Reviewer #1: Yes
Reviewer #2: No

____

4. Is the manuscript presented in an intelligible fashion and written in standard English?

PLOS ONE does not copyedit accepted manuscripts, so the language in submitted

articles must be clear, correct, and unambiguous. Any typographical or grammatical errors should be corrected at revision, so please note any specific errors here.
Reviewer #1: Yes
Reviewer #2: Yes

____

5. Review Comments to the Author

Please use the space provided to explain your answers to the questions above. You may also include additional comments for the author, including concerns about dual publication, research ethics, or publication ethics. (Please upload your review as an attachment if it exceeds 20,000 characters)
Reviewer #1: Missed opportunity for family planning counselling along the continuum of care in Arusha region, Tanzania.
Abstract
L 6&7: Associated factors of what: behavioral of the women or the health provider? What about health worker knowledge and skills, commodity supply, distance to the health facility, please clarify
Please define women of reproductive age (15-49 years of age).
We thank the reviewer for the observation, we have made the additions and defined the women of reproductive age. The authors acknowledge that apart from the women's behavioral factors studied in this paper, there could be other factors that could contribute. However, the Health worker and/or health facility related factors were not observed or studied at this time in this study project.
Methods
L 11: I suggest 'face-to-face'
We thank the reviewer for the comment, we have made the revision
L 11: Continue with to the abbreviation 'FP' after it has been introduced
We thank the reviewer for the comment, we have made the revision
L 13: The FP counselling: is it a group or one-on-one (confidential) counselling during the clinic visit?
We thank the reviewer for this important comment. FP counselling is done on a one-on-one basis between health care worker and client. Although in some instances general information on FP can be provided as group, but later on detailed information and client is given time to ask any questions or concerns.
L 16: Would contraceptive prevalence rate (CPR) or even modern CPR (mCPR) be more appropriate?
We thank the reviewer for this comment. Contraceptive Prevalence Rate (CPR) or modern CPR (mCPR) will tell us the extent or current usage of contraceptive or modern contraceptive. To determine the association between contraceptive usage and receipt of FP counselling, we implored a modified poisson regression model, whereas Prevalence Ratio (PR) was estimated.

Results
L 18: The percentage is sometime cited as '38%' and sometime as "40%" Please clarify
We thank the reviewer for the comment, we have made the revision
Conclusion
L 29: What about the other associated factors that is mentioned in the introduction. Were there any outcomes?
We thank the reviewer for the comment, we have made the revision

Introduction
L 12-19: I suggest taking above before the background reference status of FP situation in TZ
We thank the reviewer for the comment, we have made the changes
Page 4, L 13: the study also assesse'd' whether…
We thank the reviewer for the comment, we have made the revision

Materials and methods
L 16: Please stay with the abbreviation WRA
We thank the reviewer for the comment, we have made the editions in the revised manuscript
L 19: reference the data source: e.g. ministry of health or other partners
We thank the reviewer for the comment, we have added the reference

L 21: I suggest to delete "the"
We have made the deletion, thanks for the observation
Page 5, L 15: Are these referring to the "other associated factors"?
Thanks for the comment. Other associated factors include the demographics and other behavioral characteristics of the women.
L 16, L17 and L19: keep to the abbreviation introduced "FP"
We thank the reviewer for the comment, we have made the revisions
L 21: the outcome of L18-21 did not also come out in the conclusion
Thanks for the comment. The secondary outcome contraceptive usage was used to determine the association between receipt of FP counselling and current contraceptive usage. We found a significant association between receipt of FP counselling at any PNC visit and current usage of contraception (see Table 3). Conclusion was revised.
L 22: I suggest to include ‘as shown in fig 1’.
We thank the reviewer for the important observation. We have placed the figure in the data collection section, where sample selection was described.

Sample selection description flow chart
L 1: FP counselling of (1,256/3,116) is 40% not 38%.
We thank the reviewer for the comment, we have made the revisions
L7-10, please use a space before the opening bracket
If the analysis is done up this category, the coverage should be 40% instead of 38%.
We thank the reviewer for the comment, we have made the revisions

Characteristic of the study participants
L 7: This is confusing, if 462 was excluded then, does it mean analysis for coverage was done on "2,654" out of the 3,116, then coverage of 38% is still questionable.
We thank the reviewer for the comment. The 462 women who were never pregnant in the past two years, were excluded in the separate analysis that looked on predictors of FP counselling at any ANC and PNC visits. The revisions are made in the revised manuscript.

Table 1: Characteristic of the study participants (N=3,116)
This should be discussed as one of the associated factors to FP counselling coverage in the conclusion
We thank the reviewer for the comment, we have made the revisions
Please double check your subtotals, for example age=3115, marital status=3,115, work/employed only = 3114, etc
Thanks for the comment, we have double checked the subtotals. Age and marital status had one missing value in these variables, where employment status had two missing values.

Predictor of receipt of family planning counselling along the continuum of care
L 2: keep to the abbreviation "FP"
We thank the reviewer for the comment, we have made the revision
L 3: it is correctly stated here but in the Abstract, it is stated as 38%. Please don’t use the word ‘about’ so the sentence needs reframing.
We thank the reviewer for the comment, we have made the revision

Discussion
L 4: Staying with the abbreviation WRA
We have made the revision, thanks for the comment.
L 7: Please discuss further, as table 1 show combined % for poorest and poorer as 24.2%, does this mean low FP counselling coverage is found among the middle to richest category; this also goes for employment.
We thank the reviewer for the comment, we have revised the discussion to include detailed information in the revised manuscript.
L 10: here again the 38% instead of 40%
We thank the reviewer for the comment, we have made the revision

Strength and Limitations
L 5: If these factors were not assessed, then why are they mentioned in the abstract "aim" to identify the associated factors.
Thanks for the comment. The study assessed the predictors of receiving FP counselling and determined to see if there is any association between receiving of FP

Powered by Editorial Manager® and ProduXion Manager® from Aries Systems Corporation

counselling and Current contraceptive usage. However, the study did not look at the reasons why some women did not use contraception even after receiving FP counselling.
Conclusion
L 11: Here again the coverage is not consistent in the other section such as the conclusion in the abstract.
We thank the reviewer for the comment, we have made the revision

Reviewer #2:
The title refers to continuum of care while only ANC and postpartum periods are considered! Thanks for the comment. The study assessed FP counselling at any health facility visit, at any ANC visit and at any PNC visit in the past two years.

It is not clear how 38% was calculated since 3115 women were included in the analysis "Overall, 1,256 (38%) women reported that they received FP counseling at any of the health facility visits in the past two years.
We thank the reviewer for the comment, we have made the revision in the revised manuscript.

Of the 1,389 and 1,409 women who had contact with the service delivery points for ANC and PNC visits respectively; 27% and 26% had a missed FP counseling at ANC and PNC visit respectively." Calculations of 27 and 26% are also not clear!
We thank the reviewer for the comment, we have made the revision in the revised manuscript after double checking the calculations.

"The data were originally collected for an impact evaluation of a community-based intervention on contraceptive information, counselling and referral." Are the results presented in this paper before the intervention or after the intervention?
Thanks for the question. The results presented in the paper were for the baseline data before the intervention.

"A total of 3,116 women were included in the overall analysis; whereas 462 women who had no pregnancy were excluded for the ANC and PNC analysis." In figure 1, WRA included in the analysis was 3115, where did the extra participant come from to make them 3116?
Thanks for the comment. Figure one shows a total of 3,116 WRA included in the analysis (see Fig 1)

Where did the 462 women excluded come from since in figure 1, they are not shown and pregnancy was not mentioned as an exclusion criteria?
We thank the reviewer for the comment. The 462 women who were never pregnant in the past two years, were excluded in a separate sub-analysis that looked on predictors of FP counselling at any ANC and PNC visits. The clarifications are made in the revised manuscript.

"Overall, among the women who had had births in the last 30 months more than seventy percent received FP counselling at ANC and PNC visits (73% and 74% respectively)." The reported percentage in the results section are different from those in the abstract, since it is reported that only 38% received FP counselling.
We thank the reviewer for this important observation. Among the women that visited the health facility for any health-related visit in the past two years, 40% received FP counselling. Among the women who had had births in the last 30 months; 1,389 and 1,409 women had contact with the service delivery points for ANC and PNC visits respectively. Of these 68.8% and 73.6% received FP counselling at ANC and PNC visits respectively. Necessary revisions were made in the revised manuscript.

In the discussion, it is not clear what the authors are saying in this statement "These findings imply that 61% of the women missed FP counselling when they visited the facility for any health reason; while 26.8% and 26.2% missed FP counselling at 1 ANC and post-natal care (PNC) visits respectively." The discussion needs to be redone once the results have been revised.
We thank the reviewer for this important observation. Among the women that visited the health facility for any health-related visit in the past two years, only 40% received FP counselling. This means that 60% of the women that visited the health facility for

Powered by Editorial Manager® and ProduXion Manager® from Aries Systems Corporation

	any health-related visit did not get FP counselling. Among the women who had had births in the last 30 months; 1,389 and 1,409 women had contact with the service delivery points for ANC and PNC visits respectively. Of these 31.2% and 26.4% missed FP counselling at ANC and PNC visits respectively. Necessary revisions were made in the revised manuscript.____
**Additional Information:**	
**Question**	**Response**
**Financial Disclosure**<br><br>Enter a financial disclosure statement that describes the sources of funding for the work included in this submission. Review the submission guidelines for detailed requirements. View published research articles from *PLOS ONE* for specific examples.<br><br>This statement is required for submission and **will appear in the published article** if the submission is accepted. Please make sure it is accurate.<br><br>**Unfunded studies**<br>Enter: *The author(s) received no specific funding for this work.*<br><br>**Funded studies**<br>Enter a statement with the following details:<br>• Initials of the authors who received each award<br>• Grant numbers awarded to each author<br>• The full name of each funder<br>• URL of each funder website<br>• Did the sponsors or funders play any role in the study design, data collection and analysis, decision to publish, or preparation of the manuscript?<br>• **NO** - Include this sentence at the end of your statement: *The funders had no role in study design, data collection and analysis, decision to publish, or preparation of the manuscript.*<br>• **YES** - Specify the role(s) played.<br><br>* typeset	This project was made possible by a grant from an anonymous donor to Harvard T.H. Chan School of Public Health. The funder had no role in study design, data collection and analysis, decision to publish, or preparation of the manuscript.
**Competing Interests**<br><br>Use the instructions below to enter a competing interest statement for this submission. On behalf of all authors, disclose any competing interests that could be perceived to bias this	None of the research costs or authors' salaries were funded, in whole or in part, by a tobacco company. The identity of the donor is irrelevant to editors or reviewers' assessment of the validity of the work. The authors are not aware of any competing interests.

work—acknowledging all financial support and any other relevant financial or non-financial competing interests.<br><br>This statement **will appear in the published article** if the submission is accepted. Please make sure it is accurate. View published research articles from *PLOS ONE* for specific examples.<br><br>**NO authors have competing interests**<br><br>Enter: *The authors have declared that no competing interests exist.*<br><br>**Authors with competing interests**<br><br>Enter competing interest details beginning with this statement:<br><br>*I have read the journal's policy and the authors of this manuscript have the following competing interests: [insert competing interests here]*<br><br>* typeset	
**Ethics Statement**<br><br>Enter an ethics statement for this submission. This statement is required if the study involved:<br><br>• Human participants<br>• Human specimens or tissue<br>• Vertebrate animals or cephalopods<br>• Vertebrate embryos or tissues<br>• Field research<br><br>Write "N/A" if the submission does not require an ethics statement.<br><br>General guidance is provided below. Consult the submission guidelines for detailed instructions. **Make sure that all information entered here is included in the Methods section of the manuscript.**	Ethical approval was received from the National Institute of Medical Research (NIMR) - NIMRIHQIR.8cNol. 1/1171, the Kilimanjaro Christian Medical College Institutional Review Board – 2240 and Harvard T.H. Chan School of Public Health Institutional Review Board - RB17-1794 to conduct the household survey. A written participant consent was obtained before participation in the study for women aged 18 years and older. Participants who were aged 16 – 17 an assent was requested. Consent for them to participate was sought from partners for those who were married/ cohabiting, and from parents/ guardians for those who were under parental care. The women included in this study were only the ones who consented to participate in the study.

**Format for specific study types**<br><br>**Human Subject Research (involving human participants and/or tissue)**<br>• Give the name of the institutional review board or ethics committee that approved the study<br>• Include the approval number and/or a statement indicating approval of this research<br>• Indicate the form of consent obtained (written/oral) or the reason that consent was not obtained (e.g. the data were analyzed anonymously)<br><br>**Animal Research (involving vertebrate animals, embryos or tissues)**<br>• Provide the name of the Institutional Animal Care and Use Committee (IACUC) or other relevant ethics board that reviewed the study protocol, and indicate whether they approved this research or granted a formal waiver of ethical approval<br>• Include an approval number if one was obtained<br>• If the study involved *non-human primates*, add *additional details* about animal welfare and steps taken to ameliorate suffering<br>• If anesthesia, euthanasia, or any kind of animal sacrifice is part of the study, include briefly which substances and/or methods were applied<br><br>**Field Research**<br><br>Include the following details if this study involves the collection of plant, animal, or other materials from a natural setting:<br>• Field permit number<br>• Name of the institution or relevant body that granted permission	
**Data Availability**<br><br>Authors are required to make all data underlying the findings described fully available, without restriction, and from the time of publication. PLOS allows rare exceptions to address legal and ethical concerns. See the PLOS Data Policy and FAQ for detailed information.	Yes - all data are fully available without restriction

A Data Availability Statement describing where the data can be found is required at submission. Your answers to this question constitute the Data Availability Statement and **will be published in the article**, if accepted.<br><br>**Important:** Stating 'data available on request from the author' is not sufficient. If your data are only available upon request, select 'No' for the first question and explain your exceptional situation in the text box.<br><br>Do the authors confirm that all data underlying the findings described in their manuscript are fully available without restriction?	
**Describe where the data may be found in full sentences. If you are copying our sample text, replace any instances of XXX with the appropriate details.**<br><br>• If the data are **held or will be held in a public repository**, include URLs, accession numbers or DOIs. If this information will only be available after acceptance, indicate this by ticking the box below. For example: *All XXX files are available from the XXX database (accession number(s) XXX, XXX.*).<br>• If the data are all contained **within the manuscript and/or Supporting Information files**, enter the following: *All relevant data are within the manuscript and its Supporting Information files.*<br>• If neither of these applies but you are able to provide **details of access elsewhere**, with or without limitations, please do so. For example:<br><br>*Data cannot be shared publicly because of [XXX]. Data are available from the XXX Institutional Data Access / Ethics Committee (contact via XXX) for researchers who meet the criteria for access to confidential data.*<br><br>*The data underlying the results presented in the study are available from (include the name of the third party*	All relevant data are within the manuscript and its Supporting Information files.

and contact information or URL).<br>• This text is appropriate if the data are owned by a third party and authors do not have permission to share the data.<br><br>* typeset	
Additional data availability information:	

Powered by Editorial Manager® and ProduXion Manager® from Aries Systems Corporation

Manuscript Click here to access/download;Manuscript;Manuscript.doc

Missed opportunity for family planning counselling along the continuum of care in Arusha region, Tanzania

Caroline Amour*[1], Rachel Manongi[2], Michael J. Mahande[1], Bilikisu Elewonibi[4], Amina Farah[3], Sia E. Msuya[1,2,3], Iqbal Shah[4]

Affiliations

[1] Department of Epidemiology and Biostatistics, Institute of Public Health, Kilimanjaro Christian Medical University College, P.O. Box 2240, Moshi, Tanzania

[2] Department of Community Health, Institute of Public Health, Kilimanjaro Christian Medical University College, P.O. Box 2240, Moshi, Tanzania

[3] Department of Community Health, KCMC Hospital, P.O. Box 3010, Moshi, Tanzania

[4] Department of Global Health and Population, Harvard University T.H. Chan School of Public Health, Boston, Massachusetts, USA

***Corresponding author**

E-mail: lyneamour@gmail.com (CA)

Abstract

Introduction

Adequate sexual and reproductive health information is vital to women of reproductive age (WRA) 15 to 49 years, for making informed choices on their reproductive health including family planning (FP). However, many women who interact with the health system continue to miss out this vital service. The study aimed to identify the extent of provision of FP counselling at service delivery points and associated behavioral factors among women of reproductive age in two districts of Arusha region. It also determined the association between receipt of FP counselling and contraceptive usage.

Methods

Data were drawn from a cross-sectional survey of 5,208 WRA residing in two districts of Arusha region in Tanzania; conducted between January and May 2018. Multistage sampling technique was employed to select the WRA for the face-to-face interviews. FP counseling was defined as receipt of FP information by a woman during any visit at the health facility for, antenatal care (ANC), or for post-natal care (PNC). Analyses on receipt of FP counseling were done on 3,116 WRA, aged 16-44 years who were in contact with health facilities in the past two years. A modified Poisson regression model was used to determine the Prevalence Ratio (PR) as a measure of association between receipt of any FP counseling and current use of modern contraception, controlling for potential confounders.

Results

Among the women that visited the health facility for any health-related visit in the past two years, 1,256 (40%) reported that they received FP counselling. Among the women who had had births in the last 30 months; 1,389 and 1,409 women had contact with the service delivery points for ANC and PNC visits respectively. Of these 31% and 26% had a missed FP counseling at ANC and PNC visit respectively. Women who were not formally employed were more likely to receive FP counselling during facility visit than others. WRA who received any FP counseling at PNC were

significantly more likely to report current use of modern contraception than those who did not (adjusted PR [adj. PR] =1.28; 95% Confidence Interval [CI]: 1.09, 1.49).

Conclusion

Overall, only 40% women reported that they received any form of FP counseling when they interfaced with the healthcare system in the past two years. Informally employed women were more likely to receive FP counselling, and women who received FP counselling during PNC visits were significantly more likely to use contraceptive in comparison to the women who did not receive FP counselling. This presents a missed opportunity for prevention of unintended pregnancies and suggests a need for further integration of FP counseling into the ANC and PNC visits.

Key words: Missed opportunity, family planning, counselling, Tanzania

Introduction

Contraceptive counselling is a key intervention that could decrease unintended pregnancies by increasing contraceptive uptake and enabling effective contraceptive use. Contraceptive counselling has been shown to be associated with increased contraceptive use [1–4]. Several studies emphasize the importance of contraceptive counselling; it creates opportunities for a woman to obtain necessary information to make an informed decision that is appropriate for the reproductive needs and goals of the client [5–8]. Contraceptive counselling has also been shown to increase method continuation, reduce contraceptive switching/discontinuation and increase method satisfaction [9].

Compared with women in high income countries, women in low- and middle- income countries face greater risk of unwanted pregnancies and unsafe abortions which may lead to future health complications. One in three pregnancies in Tanzania are either unwanted or mistimed [10] contributing to an estimated 25,000 unsafe abortions annually. Many of these abortions occur in women under age 25 years [11]. Unsafe abortions contribute to 16% of maternal deaths [12]. Contraception is vital, given the high prevalence of unintended pregnancy and its impact on the health of the woman and child [13]. More than 30% of maternal and 10% of child mortality can be averted by the use of FP methods [14]. The most current estimate of modern contraceptive prevalence rate (mCPR) among married/ cohabiting women in Tanzania is 32% in 2015-2016 [10], well below the Tanzanian government's target of 45% target by 2020 [15].

Most women in Tanzania come in contact with the health care sector numerous times during their lifespan. According to the 2015 Tanzanian Demographic and Health Survey (TDHS), 98% of women who have had at least one pregnancy attended at least one antenatal

care (ANC) visit, 64% deliver at a health facility, and the child vaccination coverage was greater than 95% in more than 90% of the districts [10]. This creates an opportunity for FP information to be disseminated during pre-natal, post-natal, and maternal health service delivery. Provision of FP information and counseling is an essential aspect of Tanzania maternal health plan and provided free of charge [16–18]. During these visits women are expected to receive information on FP methods that are available, possible side effects, and the importance of resuming the FP after delivery [1].

There is limited information on the prevalence of contraceptive counselling in Tanzania when women come into contact with health facilities (HFs). One study found that FP counselling is more likely to be offered in private service delivery points compared to public ones [19]. In response to the Tanzania FP 2020 commitment of reaching 45% modern contraceptive prevalence rate by 2020, this study determines the extent of provision of FP counselling at service delivery points, and its associated factors in Arusha. The study also assessed whether being counselled during clinic visit increases the use of modern contraceptives.

Materials and Methods

Data collection

Data were drawn from a cross-sectional survey of WRA in two districts of Arusha region, northeastern Tanzania. The household survey data were collected from January to May 2018. The data were originally collected for an impact evaluation of a community-based intervention on contraceptive information, counselling and referral[20]. The survey was conducted on a representative sample of females aged 16-44 years, residing in the study area. A multi-stage random sampling was employed to select 3,938 women of reproductive age from Arusha city

council and Meru district council. Further details on the study, participants and sampling strategy are described elsewhere [20].Women who consented to participate in the survey were asked questions relating to pregnancy history, use of FP services, and whether or not they had ever received FP counselling at a health facility. Only women who were sexually active and had visited a health facility in the last two years were included in the analysis (Fig **1**).

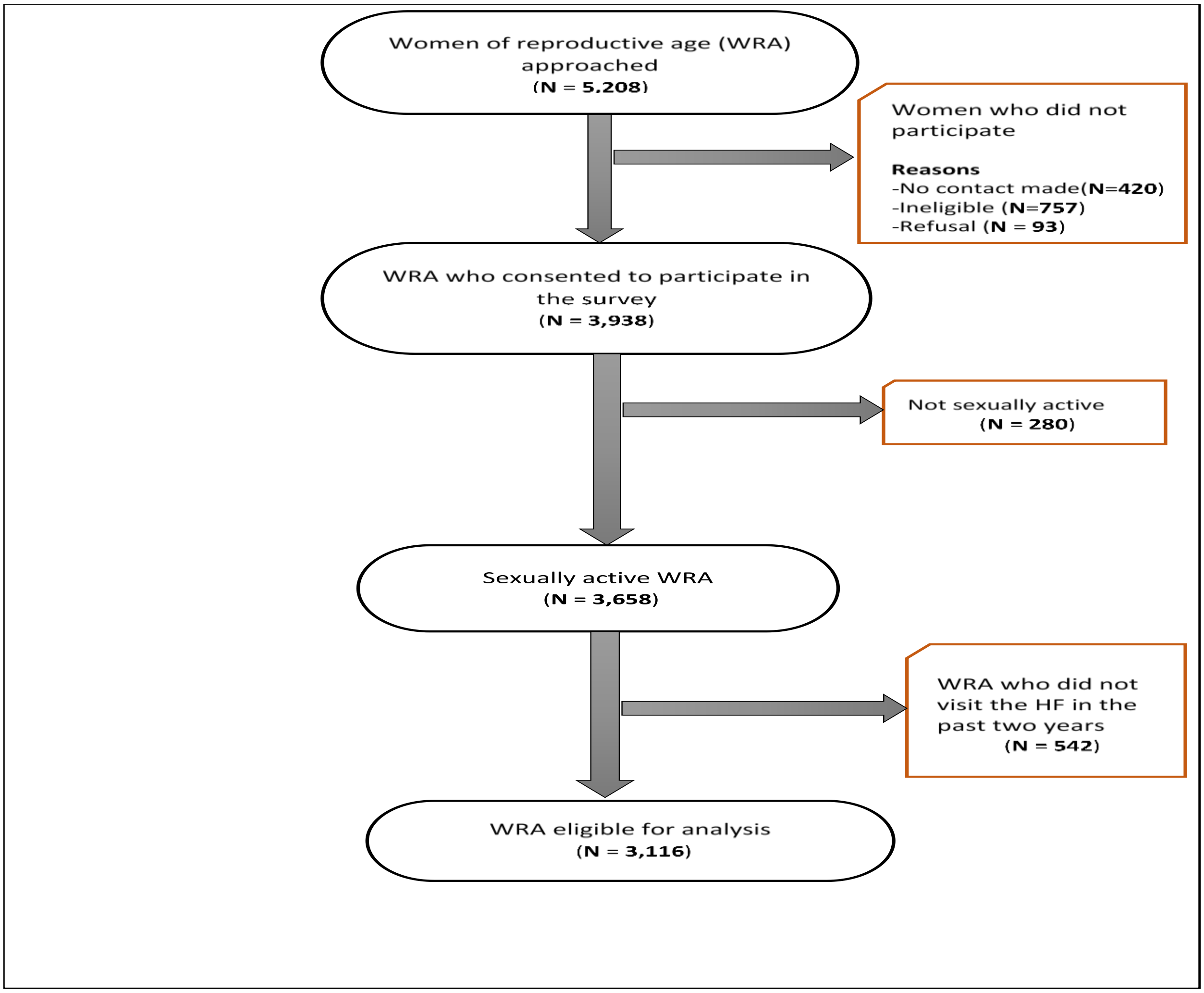

Fig 1: Sample selection description flowchart

Study variables

Outcome variable

The outcome of interest in this analysis was whether or not a woman received FP counselling during any visit to a health facility (HF), during a visit for ANC and visit for PNC within the past two years. Women were included if they had any visit, and then a separate analysis was limited to those women who had had a birth in the last 30 months and who visited for ANC or PNC. Women were considered to have received FP counseling if, at any visit to the HF within the past two years, they reported that they received and/or discussed any information about FP with a health provider. The definition of counselling used was broad and did not specify the content of what the woman and her provider discussed. A missed opportunity for FP counseling was defined as a woman's ANC or PNC clinic visit at which a staff member did not provide any FP counseling.

In this analysis, current use of modern contraceptive was the secondary study outcome and was defined as currently using any of the following methods of FP to delay or limit pregnancy i.e. female sterilization, intra-uterine device, injectables, implants, oral contraceptive pills, condoms and emergency contraception. This variable was categorized as 1 if currently using any one of the modern contraceptive methods, and 0 if otherwise.

Explanatory variables

Explanatory variables in this analysis included socio-demographic characteristics and women characteristics, from all women enrolled in the above-mentioned survey.

Women's socio-demographic information (age as a continuous variable; marital status categorized as Never married (0),Married or cohabiting (1) and Divorced/widowed/separated (2); Occupation categorized as (0) for those employed or do any work and (1) otherwise; distance to

health facility was categorized as less than 2 km (0), 2-3 km (1) and 4 km and above (2) and parity as a continuous variable were included in the analysis; with reference from other studies done on FP Counselling [14,21]. To construct the wealth index, we identified variables in our survey that are similar to the ones in the Tanzania Demographic and Health Survey (TDHS). It was created as a composite, asset-based measure from a series of questions and

then standardized to quintiles. Using these common variables, we categorized using the same cut-off points as the TDHS through principal components analysis. Hence, we ended up with five categories or groups of wealth index namely, poorest, poorer, medium, richer, and richest.

Data Analysis

Analyses were conducted on 3,116 women to provide descriptive statistics for women's socio-demographic characteristics and reproductive health characteristics. Bivariate regression analysis was used to examine predictors of FP counseling, if received during health facility visit. Multivariate regression analysis was used to determine the relationship between each predictor and the outcome of interest, controlling for the confounding effects of other factors. Modified Poisson regression was used to determine the association between receipt of FP counselling and current use of modern contraception, prevalence ratio (PR) was used as the measure of association. Modified Poisson regression directly estimates prevalence ratios and produces confidence intervals with the correct nominal coverage when individual-level data are available [22,23]. We used sample weights calculated based on the multistage sampling design in all analyses. All data were analyzed using STATA SE 15.1 software (StataCorp, USA).

Ethical Consideration

Ethical approval was received from the National Institute of Medical Research (NIMR) - NIMRlHQIR.8cNol. 1/1171, the Kilimanjaro Christian Medical College Institutional Review Board – 2240 and Harvard T.H. Chan School of Public Health Institutional Review Board - RB17-1794 to conduct the household survey. A written participant consent was obtained before participation in the study for women aged 18 years and older. Participants who were aged 16 – 17 an assent was requested. Consent for them to participate was sought from partners for those who were married/ cohabiting, and from parents/ guardians for those who were under parental care. The women included in this study were only the ones who consented to participate in the study.

Results

Characteristics of the study participants

The sociodemographic, economic and health related characteristics of the study participants are shown in Tabl**e 1**. A total of 3,116 women were included in the overall analysis. The mean (±standard deviation) age of the women was 30.3 (±6.9) years. Most of the women were between 20 and 29 years old (43%), had three or more children (40%) and were in the richer wealth quintile (46%). Three-quarter of the study participants were currently married or living with a partner (75%). Overall, among the women who had had births in the last 30 months, sixty nine percent received FP counselling at ANC and 74% at PNC visits.

1

Table 1: Characteristics of the study participants (N =3,116)

**Characteristics**	**n (%)**
Woman age (years)	
Below 20	109 (3.7)
20-29	1396 (42.8)
30-39	1239 (40.3)
40 and above	371 (13.2)
Marital status	
Never married	416 (14.6)
Married/Cohabiting	2389 (74.7)
Divorced/widowed/separated	310 (10.7)
Work/Employed	
Yes	1974 (61.7)
No	1140 (38.3)
Wealth index	
Poorest	518 (15.2)
Poorer	271 (9.0)
Middle	519 (17.6)
Richer	1438 (45.8)
Richest	370 (12.5)
Parity	
0	462 (15.2)
1	720 (22.2)
2	780 (22.8)
3 and above	1154 (39.8)
Distance to nearest health facility	
<2 km	583 (53.3)
2_3 km	255 (22.3)
4 km and above	271 (24.4)
Received FP counselling at any Health facility visit	
Yes	1256 (40.3)
No	1860 (59.7)
Received FP counselling at any ANC visit (n=1,389)	
Yes	956 (68.8)
No	433 (31.2)
Received FP counselling at any PNC visit (n=1,409)	
Yes	1037 (73.6)
No	372 (26.4)

*% is the weighted percent

Predictors of receipt of Family Planning counseling along the continuum of care

A total of 462 women who were not pregnant in the past two years were excluded for the ANC and PNC analysis. The association between respondent's characteristics and FP counselling at the health facility in the past two years is shown in Table **2**. Forty percent (1256) of women reported that they received FP counseling at any visit of the health facility in the past two years. The receipt of FP counselling at any visit differed by employment status and wealth. A higher proportion of women who were not employed received FP counselling within the past 2 years of visiting the facilities compared to others (adjusted [adj.] PR: 1.24; 95% confidence interval [CI]: 1.20, 1.50).

Table 2: Predictors for receiving FP counselling at any health facility (HF) visit (N=3,116)

		**Receipt of Family Planning counselling**		
**Characteristics**	**Counselled** n (%)	Any HF Visit (n=3,116) aPR (95% CI)	Any ANC visits (n=1,389) aPR (95% CI)	Any PNC visits (n=1,409) aPR (95% CI)
Woman age (years)				
Below 20	27 (21.3)	Ref	Ref	Ref
20-29	632 (45.3)	0.95 (0.53-1.72)	1.65 (0.57-4.76)	1.22 (0.68-2.18)
30-39	500 (38.4)	0.70 (0.37-1.29)	1.84 (0.63-5.32)	1.25 (0.69-2.29)
40 and above	97 (23.9)	0.61 (0.30-1.23)	1.81 (0.61-5.34)	1.53 (0.84-2.79)
Marital status				
Single	88 (20.1)	Ref	Ref	Ref
Married/Cohabiting	1068 (43.7)	1.27 (0.79-2.06)	0.85 (0.71-1.03)	0.84 (0.72-0.99) *
Formerly married	100 (30.0)	0.97 (0.54-1.76)	0.88 (0.67-1.14)	0.85 (0.67-1.07)
Work/Employed				

Yes	734 (35.3)	Ref	Ref	Ref
No	522 (44.3)	1.24 (1.02-1.50) *	0.93 (0.82-1.06)	0.93 (0.82-1.07)
Wealth index				
Poorest	229 (43.6)	1.19 (0.91-1.56)	1.16 (0.99-1.35)	0.99 (0.82-1.19)
Poorer	128 (41.9)	1.36 (1.02-1.80) *	1.16 (0.96-1.41)	1.14 (0.96-1.34)
Middle	217 (40.8)	1.11 (0.89-1.43)	1.08 (0.91-1.28)	1.05 (0.89-1.23)
Richer	554 (36.1)	Ref	Ref	Ref
Richest	128 (37.3)	1.19 (0.89-1.61)	1.17 (1.00-1.36) *	1.11 (0.95-1.29)
Parity				
1	339 (47.3)	Ref	Ref	Ref
2	342 (41.1)	0.95 (0.74-1.21)	0.91 (0.76-1.12)	1.04 (0.90-1.21)
3 and above	500 (41.0)	1.15 (0.88-1.50)	0.97 (0.80-1.17)	0.98 (0.81-1.20)
Distance to nearest health facility				
<2 km	232 (41.2)	Ref	Ref	Ref
2_3 km	135 (49.9)	1.19 (0.96-1.47)	0.97 (0.84-1.12)	1.07 (0.94-1.22)
4 km and above	130 (43.5)	1.02 (0.81-1.28)	0.93 (0.80-1.09)	1.04 (0.92-1.18)

Statistically significant at *P<0.05, **P< 0.001

Association between receipt of any FP counseling and current use of modern contraception

Table 3 shows the results of analysis of association between receipt of any FP counselling services and current use of modern contraceptive among women who visited a health facility in the past two years. After adjusting for woman's age, marital status and parity; the women who received any FP counselling services at a health facility visit, had a 4% higher current usage of modern contraceptive in comparison to those who did not, although this association was not statistically significant (adjusted [adj.] PR: 1.04; 95% confidence interval [CI]: 0.92, 1.16). Current use of a modern contraceptive method was 31% more prevalent among women who received FP counselling at PNC visit compared to those who didn't, this association was statistically significant (PR: 1.31; 95% confidence interval [CI]: 1.11, 1.54).

Table 3: Receipt of FP Counselling associated with current use of modern contraception (n=3,116)

	cPR (95% CI)	P-value	aPR (95% CI)	P-value
Received FP counselling at any Health facility visit				
Yes	1.48 (1.35-1.63)	<0.0001	1.04 (0.92-1.16)	0.554
No	Ref		Ref	
Received FP counselling at any ANC visit				
Yes	1.29 (1.11-1.49)	0.001	1.12 (0.97-1.28)	0.124
No	Ref		Ref	
Received FP counselling at any PNC visit				
Yes	1.45 (1.23-1.73)	<0.0001	1.31 (1.11-1.54)	0.001
No	Ref		Ref	

*adjusted by woman's age, marital status and parity

Discussion

Our study of missed opportunities for FP counseling among WRA along the continuum of care highlights a few important findings. About 61% of the women in our sample who had an interaction with the healthcare system in the past 2 years did not receive any form of contraceptive counselling. Women not formally employed and those in the poorer wealth quintile predicted receiving FP counseling during any health facility visit. Counseling during PNC period increased the prevalence of modern contraceptive use.

We found that the receipt of FP counseling was 40% among the women who attended the health facility for any health visit. Among the women who visited the health facility for any visit and had a birth in the last 30 months, 68.8% received FP counselling during ANC and 73.6% at

postnatal care visit. Availability of focused ANC and PNC care guidelines that stipulates what should be done at each visit including FP counseling may explain higher counseling reports than general Outpatient department (OPD)/ other clinic visits. These findings imply that 60% of the women missed FP counselling when they visited the facility for any health reason; while 31.2% and 26.4% missed FP counselling at ANC and post-natal care (PNC) visits respectively. Similarly findings have been reported elsewhere [8,14,16,18,24]. Kanyangara et al reported a 6% missed opportunity of FP counselling in sub-Saharan country sites. This difference from our study could be because in the other studies provision of FP counselling was reported by the facility-in-charge [25]. Overall, our findings highlight a missed opportunity for FP counselling among women, when coming into contact with a health facility. Hence, we suggest a vital need for a full-scale integration of FP counselling in any visit at the health facility along the continuum of care, especially at the PNC visits and child health services such as child immunization visit.

In Tanzania, 98% of women attend ANC care services provided by skilled provider [26]. ANC visits provides an opportunity for health worker to advice and counsel on the importance of FP. However, our study found that 31% of women were not counseled on FP at ANC and 26% at PNC visit. Those who were counseled during postnatal care visit were more likely to currently use modern contraceptive than those who were not counseled. This finding is similar to a study done in Ethiopia which found that FP planning counseling increased the chance of using postpartum family planning (PPFP) [27]. This is also similar to a study done in Kenya, where they found that contraceptive usage was 35% higher among women who had FP counselling during PNC [28,29]. Lack of association between FP counseling during ANC visits and current usage of modern contraceptive has also been observed in other East African studies[16,19]. It

maybe that after successful delivery, women are more receptive to the information that will improve their own health and that of the new baby, hence higher prevalence of contraceptive use after being counselled during postnatal period[16,19,30]. Improved uptake of maternal, newborn and child health interventions after counseling during postnatal period have also been reported in breastfeeding and vaccination[31].

We also found that married or cohabiting women were 16% less likely to receive contraceptive counselling at PNC visits compared to women who were not married. This could be explained by the providers' perceptions of women's need for family planning methods. A study in Kenya looked on provider-imposed barriers to FP use and found that providers reported to impose FP method restrictions on older women [32]. This association remained statistically significant after adjusting for other socio-demographics. These findings are contrary to a study in Ethiopia and Philippines, were receipt of contraceptive family planning was positively associated with the women's marital status; where married women were more likely to receive information or be counselled on contraceptive usage [14,21]. Studies in Tanzania and elsewhere have shown married/ cohabiting women are more likely to use contraceptives than single, divorced or separated women[14,28,33]. The difference in these studies to our study may be explained by the providers' perspective on married women usage of contraceptives. It may be that providers focus their attention to those who are at higher risk of not using the methods, thinking the other group will maintain their behavior.

Strength and Limitations

Our study addressed one of the prominent areas in numerous efforts to achieve the FP 2020 commitment aiding in reducing the unmet need for FP among WRA. This study had some

limitations. Our study cannot infer a causal relationship between the association of FP counselling and current usage of modern contraceptive. Also, some women might have some recall bias about the content of what was discussed during ANC or PNC visits and thus under-report contraceptive counseling. In addition, the results cannot be generalized to the general population in Tanzania. This is because WRA who interacted with the health care system, attended ANC and PNC services could be different from those who do not utilize these services. Another limitation is that counseling was reported by women, no observations were done. Further, health facility factors like providers knowledge and skills in counseling and FP as well as availability of supplies was not assessed. We also cannot fully explain why some women did not use contraception even after receiving FP counselling.

However, our study suggests that FP counselling during PNC visit is an important determinant of current contraceptive usage among WRA as the receipt of FP counselling during PNC visit was significantly associated with higher prevalence of current contraceptive usage.

Conclusion

Forty-percent of the women who had interacted with the service delivery points at any health visit had missed the opportunity for contraceptive counselling. Women with potential unmet need for contraceptive usage, had missed the opportunity for important information and counselling to prevent unwanted and mistimed pregnancies. It becomes even more prominent that women who received FP counselling during PNC visits were significantly more likely to use contraceptive in comparison to the women who did not receive FP counselling. Hence efforts should be directed at counselling women about the importance of contraception to avoid unwanted pregnancies. We emphasis particular recommendation on the need to enhance

integration of FP into the continuum of care, in order to increase access to FP information and education, counseling and ultimately contraceptives use among WRA.

Declarations

Abbreviations

cPR: Crude Prevalence ratio; aPR: Adjusted Prevalence ratio; CI: Confidence interval; CPR: Contraceptive prevalence rate; FP: Family planning; SSA: Sub-Saharan Africa; ANC: Antenatal Care; PNC: Post-natal care; PPFP: Post-partum Family Planning; WRA: Women of reproductive age; TDHS: Tanzania and demographic health survey; HF: Health facility; SDP: Service delivery point

Acknowledgements

The authors thank the Arusha Regional Medical Officer, District Medical Officers and other administrative staff in Arusha city and Meru district; for their cooperation and support during the study period. We also thank the study participants for their valuable participation in this study. Furthermore, we would like to thank the research assistants for their positive participation and involvement in this study.

Funding

This project was made possible by a grant from an anonymous donor to Harvard T.H. Chan School of Public Health.

Authors' contributions

BE, SEM and IS designed the study. All authors collected data. CA, and MJM conducted data analysis. CA, RM, MJM and SEM drafted the manuscript. CA RM MJM BE SEM AF and IS contributed to the final draft of the manuscript. All authors reviewed and gave their final approval to the manuscript.

Competing interest

The authors declare that they have no competing interest. None of the research costs or authors' salaries were funded, in whole or in part, by a tobacco company. The authors are not aware of any competing interests.

References

1. Lee JK, Parisi SM, Akers AY, Borrerro S, Schwarz EB. The Impact of Contraceptive Counseling in Primary Care on Contraceptive Use. Journal of General Internal Medicine. 2011;26: 731. doi:10.1007/s11606-011-1647-3

2. Ali, Mohammed, Cleland, John, Shah, Iqbal. Causes and consequences of contraceptive discontinuation: evidence from 60 demographic and health surveys. WHO; 2012. Available: https://scholar.google.com/scholar_lookup?title=Causes%20and%20consequences%20of%20contraceptive%20discontinuation%3A%20evidence%20from%2060%20demographic%20and%20health%20surveys&publication_year=2012&author=Ali%2CM&author=Cleland%2CJ&author=Shah%2CI

3. Calhoun LM, Speizer IS, Rimal R, Sripad P, Chatterjee N, Achyut P, et al. Provider imposed restrictions to clients' access to family planning in urban Uttar Pradesh, India: a mixed methods study. BMC Health Serv Res. 2013;13: 532. doi:10.1186/1472-6963-13-532

4. Hardee K, Harris S, Rodriguez M, Kumar J, Bakamjian L, Newman K, et al. Achieving the goal of the London Summit on Family Planning by adhering to voluntary, rights-based family planning: what can we learn from past experiences with coercion? Int Perspect Sex Reprod Health. 2014;40: 206–214. doi:10.1363/4020614

5. Jain AK, Hardee K. Revising the FP Quality of Care Framework in the Context of Rights-based Family Planning. Studies in Family Planning. 2018;49: 171–179. doi:10.1111/sifp.12052

6. Reddy KS, Patel V, Jha P, Paul VK, Shiva Kumar AK, Dandona L. Towards achievement of universal health care in India by 2020: a call to action. Lancet. 2011;377: 760–768. doi:10.1016/S0140-6736(10)61960-5

7. Stover J, Sonneveldt E. Progress toward the Goals of FP2020. Studies in Family Planning. 2017;48: 83–88. doi:10.1111/sifp.12014

8. Nabirye J, Matovu JKB, Bwanika JB, Makumbi F, Wanyenze RK. Missed opportunities for family planning counselling among HIV-positive women receiving HIV Care in Uganda. BMC Women's Health. 2020;20: 91. doi:10.1186/s12905-020-00942-6

9. Schivone G, Glish L. Contraceptive counseling for continuation and satisfaction. Current Opinion in Obstetrics and Gynecology. 2017;29: 443–448. doi:10.1097/GCO.0000000000000408

10. Ministry of Health, Community Development, Gender, Elderly and Children (MoHCDGEC) [Tanzania Mainland], Ministry of Health (MoH) [Zanzibar], National Bureau of Statistics (NBS), Office of the Chief Government Statistician (OCGS), ICF. Tanzania Demographic and Health Survey and Malaria Indicator Survey (TDHS-MIS). 2016 [cited 20 Dec 2018]. Available: https://dhsprogram.com/what-we-do/survey/survey-display-485.cfm

11. World Health Organization, UNICEF, United Nations, Department of Economic and Social Affairs, Population Division, World Bank. Trends in maternal mortality: 1990 to 2015 : estimates by WHO, UNICEF, UNFPA, World Bank Group and the United Nations Population Division. 2015. Available: http://www.who.int/reproductivehealth/publications/monitoring/maternal-mortality-2015/en/

12. MINISTRY OF HEALTH AND SOCIAL WELFARE. United Republic of Tanzania Ministry of Health and Social Welfare, National Family Planning Costed Implementation Program, 2010– 2015, Dar es Salaam, Tanzania: Reproductive and Child Health Section, 2010. 2010.

13. UNICEF. Maternal and child health fact sheet. Nov 2017 [cited 2 Jan 2020]. Available: https://www.unicef.org/tanzania/reports/maternal-and-child-health-fact-sheet

14. Tafere TE, Afework MF, Yalew AW. Counseling on family planning during ANC service increases the likelihood of postpartum family planning use in Bahir Dar City Administration, Northwest Ethiopia: a prospective follow up study. Contraception and Reproductive Medicine. 2018;3: 28. doi:10.1186/s40834-018-0081-x

15. FP 2020. Tanzania | Family Planning 2020 Commitment. 2013 [cited 8 Oct 2019]. Available: https://www.familyplanning2020.org/tanzania

16. Dynes MM, Bernstein E, Morof D, Kelly L, Ruiz A, Mongo W, et al. Client and provider factors associated with integration of family planning services among maternal and reproductive health clients in Kigoma Region, Tanzania: a cross-sectional study, April–July 2016. Reproductive Health. 2018;15: 152. doi:10.1186/s12978-018-0593-5

17. Kamhawi S, Underwood C, Murad H, Jabre B. Client-centered counseling improves client satisfaction with family planning visits: evidence from Irbid, Jordan. Glob Health Sci Pract. 2013;1: 180–192. doi:10.9745/GHSP-D-12-00051

18. Obare F, Liambila W, Birungi H. Family planning counselling and use among clients seeking abortion services in private health facilities in Kenya. 2014;28: 12.

19. Kakoko DC, Ketting E, Kamazima SR, Ruben R. Provision of Family Planning Services in Tanzania: A Comparative Analysis of Public and Private Facilities. African Journal of Reproductive Health / La Revue Africaine de la Santé Reproductive. 2012;16: 140–148.

20. Elewonibi B, Sato R, Manongi R, Msuya S, Shah I, Canning D. The distance-quality trade-off in women’s choice of family planning provider in North Eastern Tanzania. BMJ Global Health. 2020;5: e002149. doi:10.1136/bmjgh-2019-002149

21. Nagai M, Bellizzi S, Murray J, Kitong J, Cabral EI, Sobel HL. Opportunities lost: Barriers to increasing the use of effective contraception in the Philippines. PLOS ONE. 2019;14: e0218187. doi:10.1371/journal.pone.0218187

22. Shu D, Young JG, Toh S. Privacy-protecting estimation of adjusted risk ratios using modified Poisson regression in multi-center studies. BMC Med Res Methodol. 2019;19: 228. doi:10.1186/s12874-019-0878-6

23. Zou G. A Modified Poisson Regression Approach to Prospective Studies with Binary Data. Am J Epidemiol. 2004;159: 702–706. doi:10.1093/aje/kwh090

24. Puri MC, Moroni M, Pearson E, Pradhan E, Shah IH. Investigating the quality of family planning counselling as part of routine antenatal care and its effect on intended postpartum contraceptive method choice among women in Nepal. BMC Women’s Health. 2020;20: 29. doi:10.1186/s12905-020-00904-y

25. Kanyangarara M, Sakyi K, Laar A. Availability of integrated family planning services in HIV care and support sites in sub-Saharan Africa: a secondary analysis of national health facility surveys. Reproductive Health. 2019;16: 60. doi:10.1186/s12978-019-0713-x

26. TDHS. Tanzania Demographic and Health Survey and Malaria Indicator Survey 2015-2016. National Bureau of Statistics, Dar es Salaam, Tanzania and Rockville, Maryland, USA: MoHCDGEC, MoH, NBS, OCGS, and ICF; 2015 2016. Available: https://www.dhsprogram.com/pubs/pdf/SR233/SR233.pdf

27. Tafere TE, Afework MF, Yalew AW. Counseling on family planning during ANC service increases the likelihood of postpartum family planning use in Bahir Dar City Administration, Northwest Ethiopia: a prospective follow up study. Contracept Reprod Med. 2018;3: 1–9. doi:10.1186/s40834-018-0081-x

28. Achwoka D, Pintye J, McGrath CJ, Kinuthia J, Unger JA, Obudho N, et al. Uptake and correlates of contraception among postpartum women in Kenya: results from a national cross-sectional survey. Contraception. 2018;97: 227–235. doi:10.1016/j.contraception.2017.10.001

29. Do M, Kurimoto N. Women's Empowerment and Choice of Contraceptive Methods in Selected African Countries. IPSRH. 2012;38: 023–033. doi:10.1363/3802312

30. Dehingia N, Dixit A, Averbach S, Choudhry V, Dey A, Chandurkar D, et al. Family planning counseling and its associations with modern contraceptive use, initiation, and continuation in rural Uttar Pradesh, India. Reproductive Health. 2019;16: 178. doi:10.1186/s12978-019-0844-0

31. Maonga AR, Mahande MJ, Damian DJ, Msuya SE. Factors Affecting Exclusive Breastfeeding among Women in Muheza District Tanga Northeastern Tanzania: A Mixed Method Community Based Study. Matern Child Health J. 2016;20: 77–87. doi:10.1007/s10995-015-1805-z

32. Tumlinson K, Okigbo CC, Speizer IS. Provider barriers to family planning access in urban Kenya. Contraception. 2015;92: 143–151. doi:10.1016/j.contraception.2015.04.002

33. Clements S, Madise N. Who Is Being Served Least by Family Planning Providers? A Study of Modern Contraceptive Use in Ghana, Tanzania and Zimbabwe. African Journal of Reproductive Health / La Revue Africaine de la Santé Reproductive. 2004;8: 124–136. doi:10.2307/3583186

 Click here to access/download;Figure;Fig 1.tif

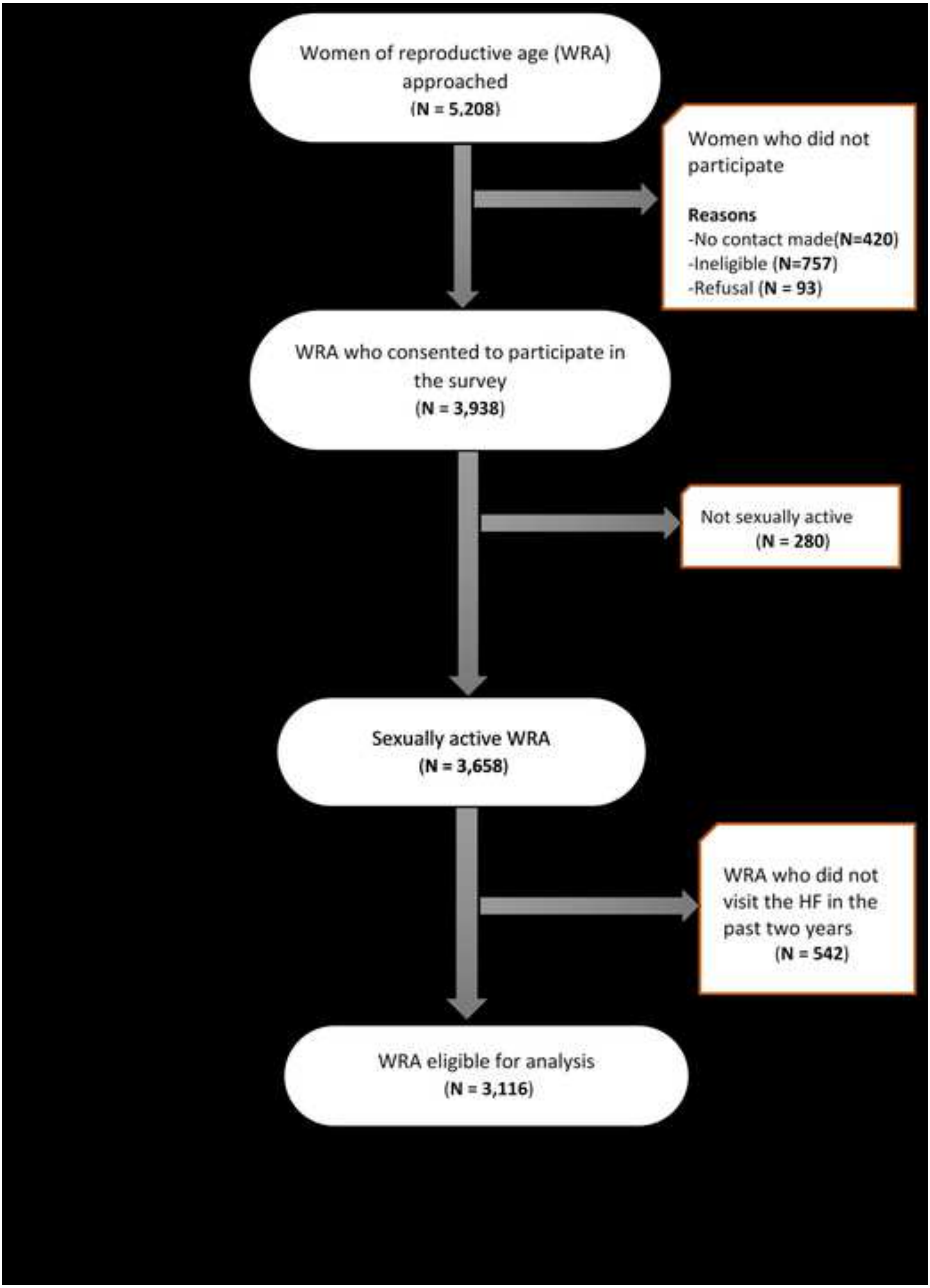

Data Underlying the study

Click here to access/download
Supporting Information
Data_FPcounselling_2021.dta

Missed opportunity for family planning counselling along the continuum of care in Arusha region, Tanzania

Style Definition: Heading 2: Font: 16 pt, Not Italic

Formatted: Heading 1, Line spacing: single

Caroline Amour*[1], Rachel Manongi[2], Michael J. Mahande[1], Bilikisu Elewonibi[4], Amina Farah[3], Sia E. Msuya[1,2,3], Iqbal Shah[4]

Formatted: Line spacing: Double

Affiliations

[1] Department of Epidemiology and Biostatistics, Institute of Public Health, Kilimanjaro Christian Medical University College, P.O. Box 2240, Moshi, Tanzania

[2] Department of Community Health, Institute of Public Health, Kilimanjaro Christian Medical University College, P.O. Box 2240, Moshi, Tanzania

[3] Department of Community Health, KCMC Hospital, P.O. Box 3010, Moshi, Tanzania

[4] Department of Global Health and Population, Harvard University T.H. Chan School of Public Health, Boston, Massachusetts, USA

***~~Correspondence~~Corresponding author**

E-mail: ~~Caroline Amour (email~~: lyneamour@gmail.com ~~(CA))~~

~~**E-mail addresses of authors:**~~

~~CA: lyneamour@gmail.com~~

RM: naeraheli@gmail.com

MJM: jmmahande@gmail.com

BE: elewonibi@hsph.harvard. Edu

AF: amifa2001@gmail.com

SEM: siaemmanuelimsuya@gmail.com

IS: ishah@hsph.harvard.edu

Abstract

Introduction

Adequate sexual and reproductive health information is vital to women of reproductive age (WRA) 15 to 49 years, for making informed choices on their reproductive health including family planning (FP). However, many women who interact with the health system continue to miss out this vital service. The study aimed to identify the extent of provision of ~~family planning~~FP counselling at service delivery points and associated behavioral factors among women of reproductive age in two districts of Arusha region. It also determined the association between receipt of FP counselling and contraceptive usage.

Methods

Data were drawn from a cross-sectional survey of 5,208 WRA residing in two districts of Arusha region in Tanzania; conducted between January and May 2018. Multistage sampling technique was employed to select the WRA for the ~~face to face~~face-to-face interviews. ~~Family planning~~FP counseling was defined as receipt of FP information by a woman during any visit at the health facility for, antenatal care (ANC), or for post-natal care (PNC). Analyses on receipt of FP counseling were done on 3,116 WRA, aged 16-44 years who were in contact with health facilities in the past two years. A modified Poisson regression model was used to determine the Prevalence Ratio (PR) as a measure of association between receipt of any FP counseling and current use of modern contraception, controlling for potential confounders.

Results

Among the women that visited the health facility for any health-related visit in the past two years, 1,256 (40%) reported that they received FP counselling. Among the women who had had births in the last 30 months; 1,389 and 1,409 women had contact with the service delivery points for ANC and PNC visits respectively. ~~Overall, 1,256 (38%) women reported that they received FP counseling at any of the health facility visits in the past two years. Of the 1,389 and 1,409 women who had contact with the service delivery points for ANC and PNC visits respectively; 27~~Of these 31% and

26% had a missed FP counseling at ANC and PNC visit respectively. Women who were not formally employed were more likely to receive FP counselling during facility visit than others. WRA who received any FP counseling at PNC were significantly more likely to report current use of modern contraception than those who did not (adjusted PR [adj. PR] =1.28; 95% Confidence Interval [CI]: 1.09, 1.49).

Conclusion

Overall, only 3840% women reported that they received any form of FP counseling when they interfaced with the healthcare system in the past two years. Informally employed women were more likely to receive FP counselling, and women who received FP counselling during PNC visits were significantly more likely to use contraceptive in comparison to the women who did not receive FP counselling. This presents a missed opportunity for prevention of unintended pregnancies and suggests a need for further the integration of FP counseling into the continuum of care at allANC and PNC visits.

Key words: Missed opportunity, family planning, counselling, Tanzania

Introduction

Contraceptive counselling is a key intervention that could decrease unintended pregnancies by increasing contraceptive uptake and enabling effective contraceptive use. Contraceptive counselling has been shown to be associated with increased contraceptive use [1–4]. Several studies emphasize the importance of contraceptive counselling; it creates opportunities for a woman to obtain necessary information to make an informed decision that is appropriate for the reproductive needs and goals of the client [5–8]. Contraceptive counselling has also been shown to increase method continuation, reduce contraceptive switching/discontinuation and increase method satisfaction [9].

Compared with women in high income countries, women in low- and middle- income countries face greater risk of unwanted pregnancies and unsafe abortions which may lead to future health complications. One in three pregnancies in Tanzania are either unwanted or mistimed [10] contributing to an estimated 25,000 unsafe abortions annually. Many of these abortions occur in women under age 25 years [11]. Unsafe abortions contribute to 16% of maternal deaths [12]. Contraception is vital, given the high prevalence of unintended pregnancy and its impact on the health of the woman and child [13]. More than 30% of maternal and 10% of child mortality can be averted by the use of ~~family planning~~FP methods [14]. The most current estimate of modern contraceptive prevalence rate (mCPR) among married/ cohabiting women in Tanzania is 32% in 2015-2016 [10], well below the Tanzanian government's target of 45% target by 2020 [15].

~~Contraceptive counselling is a key intervention that could decrease unintended pregnancies by increasing contraceptive uptake and enabling effective contraceptive use. Contraceptive counselling has been shown to be associated with increased contraceptive use [7–~~

~~10]. Several studies emphasize the importance of contraceptive counselling; it creates opportunities for a woman to obtain necessary information to make an informed decision that is appropriate for the reproductive needs and goals of the client [11–13]. Contraceptive counselling has also been shown to increase method continuation, reduce contraceptive switching/discontinuation and increase method satisfaction [14].~~

Most women in Tanzania come in contact with the health care sector numerous times during their lifespan. According to the 2015 Tanzanian Demographic and Health Survey (TDHS), 98% of women who have had at least one pregnancy attended at least one antenatal care (ANC) visit, 64% deliver at a health facility, and the child vaccination coverage was greater than 95% in more than 90% of the districts [10]. This creates an opportunity for ~~family planning~~FP information to be disseminated during pre-natal, post-natal, and maternal health service delivery. Provision of ~~family planning~~FP information and counseling is an essential aspect of Tanzania maternal health plan and provided free of charge [16–18]. During these visits women are expected to receive information on ~~family planning~~FP methods that are available, possible side effects, and the importance of resuming the ~~family planning~~FP after delivery [1].

There is limited information on the prevalence of contraceptive counselling in Tanzania when women come into contact with health facilities (HFs). One study found that ~~family planning~~FP counselling is more likely to be offered in private service delivery points compared to public ones [19]. In response to the Tanzania FP 2020 commitment of reaching 45% modern contraceptive prevalence rate by 2020, this study determines the extent of provision of FP counselling at service delivery points, and its associated factors in Arusha. The study also ~~assesses~~ assessed whether being counselled during clinic visit increases the use of modern contraceptives.

Materials and Methods

Formatted: Line spacing: Double

Data collection

Formatted: Heading 2, Left, Line spacing: single

Data were drawn from a cross-sectional survey of ~~women of reproductive age~~WRA in two districts of Arusha region, northeastern Tanzania. The household survey data were collected from January to May 2018. The data were originally collected for an impact evaluation of a community-based intervention on contraceptive information, counselling and referral[20]. The survey was conducted on a representative sample of ~~women~~ females aged 16-44 years, residing in the study area. A multi-stage random sampling was employed to select ~~the~~ 3,938 women of reproductive age from Arusha city council and Meru district council. Further details on the study, participants and sampling strategy are described elsewhere [20].Women who consented to participate in the survey were asked questions relating to pregnancy history, use of ~~family planning~~FP services, and whether or not they had ever received ~~family planning~~FP counselling at a health facility. Only women who were sexually active and had visited a health facility in the last two years were included in the analysis (Figure **1**).

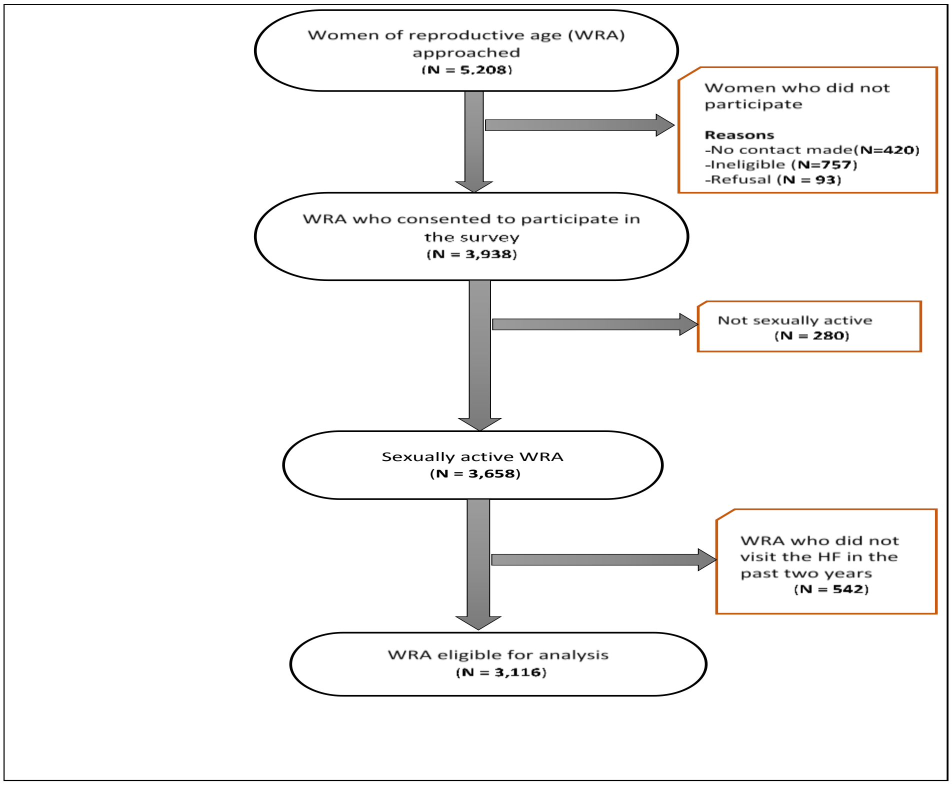

Fig 1: Sample selection description flowchart

Formatted: Line spacing: Double

Study variables

Formatted: Heading 2, Left, Line spacing: single, Adjust space between Latin and Asian text, Adjust space between Asian text and numbers

Outcome variable

The outcome of interest in this analysis was whether or not a woman received ~~family planning~~FP counselling during any visit to a health facility (HF), during a visit for ANC and visit for PNC within the past two years. Women were included if they had any visit, and then a separate analysis was limited to those women who had had a birth in the last 30 months and who visited

for ANC or PNC. Women were considered to have received FP counseling if, at any visit to the HF within the past two years, they reported that they received and/or discussed any information about FP with a health provider. The definition of counselling used was broad and did not specify the content of what the woman and her provider discussed. A missed opportunity for ~~family planning~~FP counseling was defined as a woman's ANC or PNC clinic visit at which a staff member did not provide any ~~family planning~~FP counseling.

In this analysis, current use of modern contraceptive was the secondary study outcome and was defined as currently using any of the following methods of ~~family planning~~FP to delay or limit pregnancy i.e. female sterilization, intra-uterine device, injectables, implants, oral contraceptive pills, condoms and emergency contraception. This variable was categorized as 1 if currently using any one of the modern contraceptive methods, and 0 if otherwise.

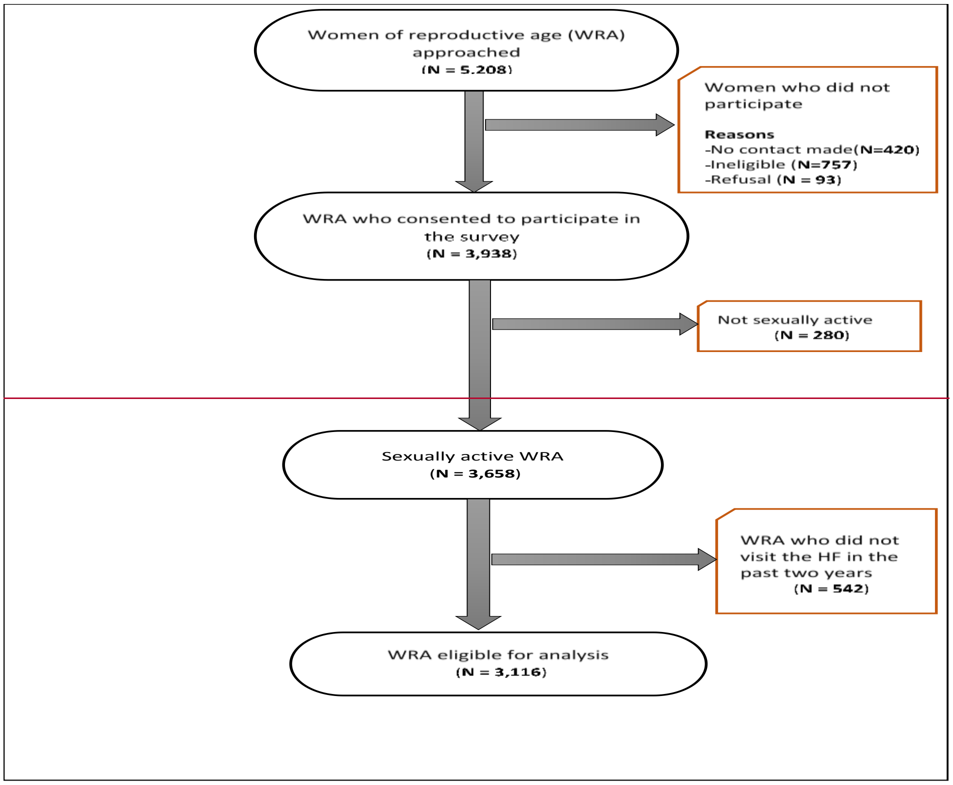

~~**Figure 1: Sample selection description flowchart**~~

Formatted: Line spacing: Double

Explanatory variables

Formatted: Heading 3, Left, Line spacing: single, Adjust space between Latin and Asian text, Adjust space between Asian text and numbers

Explanatory variables in this analysis included socio-demographic characteristics and women characteristics, from all women enrolled in the above-mentioned survey.

Women's socio-demographic information (age as a continuous variable; marital status categorized as Never married (0),Married or cohabiting (1) and Divorced/widowed/separated (2); Occupation categorized as (0) for those employed or do any work and (1) otherwise; wealth),

distance to health facility was categorized as less than 2 km (0), 2-3 km (1) and 4 km and above (2) and parity as a continuous variable were included in the analysis; with reference from other studies done on FP Counselling [14,21]. To construct the wealth index, we identified variables in our survey that are similar to the ones in the Tanzania Demographic and Health Survey (TDHS). It was created as a composite, asset-based measure from a series of questions and then standardized to quintiles. Using these common variables, we categorized using the same cut-off points as the TDHS through principal components analysis. Hence, we ended up with five categories or groups of wealth index namely, poorest, poorer, medium, richer, and richest.

Data Analysis

Analyses were conducted on 3,11~~6~~6 women to provide descriptive statistics for women's socio-demographic characteristics and reproductive health characteristics. Bivariate regression analysis was used to examine predictors of FP counseling, if received during health facility visit. Multivariate regression analysis was used to determine the relationship between each predictor and the outcome of interest, controlling for the confounding effects of other factors. Modified Poisson regression was used to determine the association between receipt of FP counselling and current use of modern contraception, prevalence ratio (PR) was used as the measure of association. Modified Poisson regression directly estimates prevalence ratios and produces confidence intervals with the correct nominal coverage when individual-level data are available [22,23]. We used sample weights calculated based on the multistage sampling design in all analyses. All data were analyzed using STATA SE 15.1 software (StataCorp, USA).

Formatted: Heading 2, Line spacing: single, Adjust space between Latin and Asian text, Adjust space between Asian text and numbers

Ethical Consideration

Formatted: Heading 2, Left, Line spacing: single, Adjust space between Latin and Asian text, Adjust space between Asian text and numbers

Ethical approval was received from the National Institute of Medical Research (NIMR) - NIMRlHQIR.8cNol. 1/1171, the Kilimanjaro Christian Medical College Institutional Review Board – 2240 and Harvard T.H. Chan School of Public Health Institutional Review Board - RB17-1794 to conduct the household survey. A written participant consent was obtained before participation in the study for women aged 18 years and older. Participants who were aged 16 – 17 an assent was requested. Consent for them to participate was sought from partners for those who were married/ cohabiting, and from parents/ guardians for those who were under parental care. The women included in this study were only the ones who consented to participate in the study.

Formatted: Line spacing: Double

Results

Characteristics of the study participants

Formatted: Heading 2, Left, Line spacing: single, Adjust space between Latin and Asian text, Adjust space between Asian text and numbers

The sociodemographic, economic and health related characteristics of the study participants are shown in Tabl**e 1**. A total of 3,116 women were included in the overall analysis~~; whereas 462 women who had no pregnancy were excluded for the ANC and PNC analysis~~. The mean (±standard deviation) age of the women was 30.3 (±6.9) years. Most of the women were between 20 and 29 years old (43%), had three or more children (40%) and were in the richer wealth quintile (46%). Three-quarter of the study participants were currently married or living with a partner (75%). Sixty-one percent had primary education and below. About half of women resided in Arusha region for more than five years (52%). Overall, among the women who had

had births in the last 30 months, ~~more than seventy~~sixty nine percent received FP counselling at ANC and 74% at PNC visits ~~(73% and 74% respectively)~~.

Formatted: Line spacing: Double

Table 1: Characteristics of the study participants (N =3,116)

Characteristics	n (%)
Woman age (years)	
Below 20	109 (3.7)
20-29	1396 (42.8)
30-39	1239 (40.3)
40 and above	371 (13.2)
Marital status	
Never married	416 (14.6)
Married/Cohabiting	2389 (74.7)
Divorced/widowed/separated	310 (10.7)
Work/Employed	
Yes	1974 (61.7)
No	1140 (38.3)
Wealth index	
Poorest	518 (15.2)
Poorer	271 (9.0)
Middle	519 (17.6)
Richer	1438 (45.8)
Richest	370 (12.5)
Parity	
0	462 (15.2)
1	720 (22.2)
2	780 (22.8)
3 and above	1154 (39.8)
Distance to nearest health facility	
<2 km	583 (53.3)
2_3 km	255 (22.3)
4 km and above	271 (24.4)
Received FP counselling at any Health facility visit	
Yes	1256 (~~38.7~~40.3)
No	1860 (~~61.3~~59.7)
Received FP counselling at any ANC visit (n=1,389)	
Yes	956 (~~73.2~~68.8)
No	433 (~~26.8~~31.2)
Received FP counselling at any PNC visit (n=1,409)	
Yes	1037 (73.~~8~~6)
No	372 (26.~~2~~4)

*% is the weighted percent

Predictors of receipt of Family Planning counseling along the continuum of care

A total of 462 women who were not pregnant in the past two years were excluded for the ANC and PNC analysis. The association between respondent's characteristics and ~~family planning~~FP counselling at the health facility in the past two years is shown in Table **2**. ~~About 40%~~Forty percent (1256) of women reported that they received FP counseling at any visit of the health facility in the past two years. The receipt of FP counselling at any visit differed by employment status and wealth. A higher proportion of women who were not employed received FP counselling within the past 2 years of visiting the facilities compared to others (adjusted [adj.] PR: 1.24; 95% confidence interval [CI]: 1.20, 1.50).

Formatted: Line spacing: Double

Formatted: Heading 2, Left, Line spacing: single

Table 2: Predictors for receiving FP counselling at any health facility (HF) visit (N=3,116)

Characteristics	Counselled n (%)	Receipt of Family Planning counselling: Any HF Visit (n=3,116) aPR (95% CI)	Any ANC visits (n=1,389) aPR (95% CI)	Any PNC visits (n=1,409) aPR (95% CI)
Woman age (years)				
Below 20	27 (21.3)	Ref	Ref	Ref
20-29	632 (45.3)	0.95 (0.53-1.72)	1.65 (0.57-4.76)	1.22 (0.68-2.18)
30-39	500 (38.4)	0.70 (0.37-1.29)	1.84 (0.63-5.32)	1.25 (0.69-2.29)
40 and above	97 (23.9)	0.61 (0.30-1.23)	1.81 (0.61-5.34)	1.53 (0.84-2.79)
Marital status				
Single	88 (20.1)	Ref	Ref	Ref

Married/Cohabiting	1068 (43.7)	1.27 (0.79-2.06)	0.85 (0.71-1.03)	0.84 (0.72-0.99) *
Formerly married	100 (30.0)	0.97 (0.54-1.76)	0.88 (0.67-1.14)	0.85 (0.67-1.07)
Work/Employed				
Yes	734 (35.3)	Ref	Ref	Ref
No	522 (44.3)	1.24 (1.02-1.50) *	0.93 (0.82-1.06)	0.93 (0.82-1.07)
Wealth index				
Poorest	229 (43.6)	1.19 (0.91-1.56)	1.16 (0.99-1.35)	0.99 (0.82-1.19)
Poorer	128 (41.9)	1.36 (1.02-1.80) *	1.16 (0.96-1.41)	1.14 (0.96-1.34)
Middle	217 (40.8)	1.11 (0.89-1.43)	1.08 (0.91-1.28)	1.05 (0.89-1.23)
Richer	554 (36.1)	Ref	Ref	Ref
Richest	128 (37.3)	1.19 (0.89-1.61)	1.17 (1.00-1.36) *	1.11 (0.95-1.29)
Parity				
1	339 (47.3)	Ref	Ref	Ref
2	342 (41.1)	0.95 (0.74-1.21)	0.91 (0.76-1.12)	1.04 (0.90-1.21)
3 and above	500 (41.0)	1.15 (0.88-1.50)	0.97 (0.80-1.17)	0.98 (0.81-1.20)
Distance to nearest health facility				
<2 km	232 (41.2)	Ref	Ref	Ref
2_3 km	135 (49.9)	1.19 (0.96-1.47)	0.97 (0.84-1.12)	1.07 (0.94-1.22)
4 km and above	130 (43.5)	1.02 (0.81-1.28)	0.93 (0.80-1.09)	1.04 (0.92-1.18)

Statistically significant at *P<0.05, **P< 0.001

Association between receipt of any FP counseling and current use of modern contraception

Table 3 shows the results of analysis of association between receipt of any FP counselling services and current use of modern contraceptive among women who visited a health facility in the past two years. After adjusting for woman's age, marital status and parity; the women who received any FP counselling services at a health facility visit, had a 4% higher current usage of modern contraceptive in comparison to those who did not, although this association was not statistically significant (adjusted [adj.] PR: 1.04; 95% confidence interval [CI]: 0.92, 1.16). Current use of a modern contraceptive method was 31% more prevalent among women who received FP counselling at PNC visit compared to those who didn't, this association was statistically significant (PR: 1.31; 95% confidence interval [CI]: 1.11, 1.54).

Table 3: Receipt of FP Counselling associated with current use of modern contraception (n=3,116)

Formatted: Line spacing: Double

	cPR (95% CI)	P-value	aPR (95% CI)	P-value
Received FP counselling at any Health facility visit				
Yes	1.48 (1.35-1.63)	<0.0001	1.04 (0.92-1.16)	0.554
No	Ref		Ref	
Received FP counselling at any ANC visit				
Yes	1.29 (1.11-1.49)	0.001	1.12 (0.97-1.28)	0.124
No	Ref		Ref	
Received FP counselling at any PNC visit				
Yes	1.45 (1.23-1.73)	<0.0001	1.31 (1.11-1.54)	0.001
No	Ref		Ref	

*adjusted by woman's age, marital status and parity

Discussion

Formatted: Line spacing: Double

Our study of missed opportunities for FP counseling among women of reproductive age (WRA) along the continuum of care highlights a few important findings. About 61% of the women in our sample who had an interaction with the healthcare system in the past 2 years did not receive any form of contraceptive counselling. Employment Women not formally employed and those in the poorer wealth quintile predicted receiving FP counseling during any clinic health facility visit. Counseling during PNC period increased the prevalence of modern contraceptive use.

We found that the receipt of FP counseling was 38.940% among the women who attended the health facility for any health visit. Among the women who visited the health facility for any visit and had a birth in the last 30 months, 73.268.8% received FP counselling during

ANC and 73.~~8~~6% at postnatal care visit. Availability of focused ANC and PNC care guidelines that stipulates what should be done at each visit including FP counseling may explain higher counseling reports than general Outpatient department (OPD)/ other clinic visits. These findings imply that ~~61~~60% of the women missed FP counselling when they visited the facility for any health reason; while ~~26.8~~31.2% and 26.~~2~~4% missed FP counselling at ANC and post-natal care (PNC) visits respectively. Similarly findings have been reported elsewhere [8,14,16,18,24]. Kanyangara et al reported a 6% missed opportunity of FP counselling in sub-Saharan country sites. This difference from our study could be because in the other studies provision of FP counselling was reported by the facility-in-charge [25]. Overall, our findings highlight a missed opportunity for FP counselling among women, when coming into contact with a health facility. Hence, we suggest a vital need for a full-scale integration of FP counselling in any visit at the health facility along the continuum of care, especially at the PNC visits and child health services such as child immunization visit.

In Tanzania, 98% of women attend ANC care services provided by skilled provider [26]. ANC visits provides an opportunity for health worker to advice and counsel on the importance of FP. However, our study found that ~~nearly 26~~31% of women were not counseled on FP at ANC ~~or~~ and 26% at PNC visit. Those who were counseled during postnatal care visit were more likely to currently use modern contraceptive than those who were not counseled. This finding is similar to a study done in Ethiopia which found that FP planning counseling increased the chance of using postpartum family planning (PPFP) [27]. This is also similar to a study done in Kenya, where they found that contraceptive usage was 35% higher among women who had FP counselling during PNC [28,29]. Lack of association between FP counseling during ANC visits and current usage of modern contraceptive has also been observed in other East African studies[16,19]. It

maybe that after successful delivery, women are more receptive to the information that will improve their own health and that of the new baby, hence higher prevalence of contraceptive use after being counselled during postnatal period[16,19,30]. Improved uptake of maternal, newborn and child health interventions after counseling during postnatal period have also been reported in breastfeeding and vaccination[31].

We also found that married or cohabiting women were 16% less likely to receive contraceptive counselling at PNC visits compared to women who were not married. This could be explained by the providers' perceptions of women's need for family planning methods. A study in Kenya looked on provider-imposed barriers to FP use and found that providers reported to impose FP method restrictions on older women [32]. This association remained statistically significant after adjusting for other socio-demographics. These findings are contrary to a study in Ethiopia and Philippines, were receipt of contraceptive family planning was positively associated with the women's marital status; where married women were more likely to receive information or be counselled on contraceptive usage [14,21]. Studies in Tanzania and elsewhere have shown married/ cohabiting women are more likely to use contraceptives than single, divorced or separated women[14,28,33]. The difference in these studies to our study may be explained by the providers' perspective on married women usage of contraceptives. It may be that providers focus their attention to those who are at higher risk of not using the methods, thinking the other group will maintain their behavior.

Strength and Limitations

Formatted: Line spacing: Double

Our study addressed one of the prominent areas in numerous efforts to achieve the FP 2020 commitment aiding in reducing the unmet need for FP among WRA. This study had some

limitations. Our study cannot infer a causal relationship between the association of FP counselling and current usage of modern contraceptive. Also, some women might have some recall bias about the content of what was discussed during ANC or PNC visits and thus under-report contraceptive counseling. In addition, the results cannot be generalized to the general population in Tanzania. This is because WRA who interacted with the health care system, attended ANC and PNC services could be different from those who do not utilize these services. Another limitation is that counseling was reported by women, no observations were done. Further, health facility factors like providers knowledge and skills in counseling and FP as well as availability of supplies was not assessed. We also cannot fully explain why some women did not use contraception even after receiving FP counselling.

However, our study suggests that FP counselling during PNC visit is an important determinant of current contraceptive usage among WRA as the receipt of FP counselling during PNC visit was significantly associated with higher prevalence of current contraceptive usage.

Conclusion

Formatted: Line spacing: Double

~~Almost ~~F~~f~~orty-percent of the women who had interacted with the service delivery points at any health visit had missed the opportunity for contraceptive counselling. Women with potential unmet need for contraceptive usage, had missed the opportunity for important information and counselling to prevent unwanted and mistimed pregnancies. It becomes even more prominent that women who received FP counselling during PNC visits were significantly more likely to use contraceptive in comparison to the women who did not receive FP counselling. Hence efforts should be directed at counselling women about the importance of contraception to avoid unwanted pregnancies. We emphasis particular recommendation on the need to enhance

integration of FP into the continuum of care, in order to increase access to FP information and education, counseling and ultimately contraceptives use among WRA.

Declarations

Formatted: Line spacing: Double

Abbreviations

Formatted: Heading 2, Left, Line spacing: single

Formatted: Line spacing: Double

cPR: Crude Prevalence ratio; aPR: Adjusted Prevalence ratio; CI: Confidence interval; CPR: Contraceptive prevalence rate; FP: Family planning; SSA: Sub-Saharan Africa; ANC: Antenatal Care; PNC: Post-natal care; PPFP: Post-partum Family Planning; WRA: Women of reproductive age; TDHS: Tanzania and demographic health survey; HF: Health facility; SDP: Service delivery point

Acknowledgements

Formatted: Heading 2, Left, Line spacing: single

Formatted: Line spacing: Double

The authors thank the Arusha Regional Medical Officer, District Medical Officers and other administrative staff in Arusha city and Meru district; for their cooperation and support during the study period. We also thank the study participants for their valuable participation in this study. Furthermore, we would like to thank the research assistants for their positive participation and involvement in this study.

Funding

Formatted: Heading 2, Left, Line spacing: single

Formatted: Line spacing: Double

This project was made possible by a grant from an anonymous donor to Harvard T.H. Chan School of Public Health.

Authors' contributions

Formatted: Heading 2, Left, Line spacing: single

BE, SEM and IS designed the study. All authors collected data. CA, and MJM conducted data analysis. CA, RM, MJM and SEM drafted the manuscript. CA RM MJM BE SEM AF and IS contributed to the final draft of the manuscript. All authors reviewed and gave their final approval to the manuscript.

Formatted: Line spacing: Double

Competing interest

Formatted: Heading 2, Left, Line spacing: single

The authors declare that they have no competing interest. None of the research costs or authors' salaries were funded, in whole or in part, by a tobacco company. The authors are not aware of any competing interests.

Formatted: Line spacing: Double

References

1. Lee JK, Parisi SM, Akers AY, Borrerro S, Schwarz EB. The Impact of Contraceptive Counseling in Primary Care on Contraceptive Use. Journal of General Internal Medicine. 2011;26: 731. doi:10.1007/s11606-011-1647-3

2. Ali, Mohammed, Cleland, John, Shah, Iqbal. Causes and consequences of contraceptive discontinuation: evidence from 60 demographic and health surveys. WHO; 2012. Available: https://scholar.google.com/scholar_lookup?title=Causes%20and%20consequences%20of%20contraceptive%20discontinuation%3A%20evidence%20from%2060%20demographic%20and%20health%20surveys&publication_year=2012&author=Ali%2CM&author=Cleland%2CJ&author=Shah%2CI

3. Calhoun LM, Speizer IS, Rimal R, Sripad P, Chatterjee N, Achyut P, et al. Provider imposed restrictions to clients' access to family planning in urban Uttar Pradesh, India: a mixed methods study. BMC Health Serv Res. 2013;13: 532. doi:10.1186/1472-6963-13-532

4. Hardee K, Harris S, Rodriguez M, Kumar J, Bakamjian L, Newman K, et al. Achieving the goal of the London Summit on Family Planning by adhering to voluntary, rights-based family planning: what can we learn from past experiences with coercion? Int Perspect Sex Reprod Health. 2014;40: 206–214. doi:10.1363/4020614

5. Jain AK, Hardee K. Revising the FP Quality of Care Framework in the Context of Rights-based Family Planning. Studies in Family Planning. 2018;49: 171–179. doi:10.1111/sifp.12052

6. Reddy KS, Patel V, Jha P, Paul VK, Shiva Kumar AK, Dandona L. Towards achievement of universal health care in India by 2020: a call to action. Lancet. 2011;377: 760–768. doi:10.1016/S0140-6736(10)61960-5

7. Stover J, Sonneveldt E. Progress toward the Goals of FP2020. Studies in Family Planning. 2017;48: 83–88. doi:10.1111/sifp.12014

8. Nabirye J, Matovu JKB, Bwanika JB, Makumbi F, Wanyenze RK. Missed opportunities for family planning counselling among HIV-positive women receiving HIV Care in Uganda. BMC Women's Health. 2020;20: 91. doi:10.1186/s12905-020-00942-6

9. Schivone G, Glish L. Contraceptive counseling for continuation and satisfaction. Current Opinion in Obstetrics and Gynecology. 2017;29: 443–448. doi:10.1097/GCO.0000000000000408

10. Ministry of Health, Community Development, Gender, Elderly and Children (MoHCDGEC) [Tanzania Mainland], Ministry of Health (MoH) [Zanzibar], National Bureau of Statistics (NBS), Office of the Chief Government Statistician (OCGS), ICF. Tanzania Demographic and Health Survey and Malaria Indicator Survey (TDHS-MIS). 2016 [cited 20 Dec 2018]. Available: https://dhsprogram.com/what-we-do/survey/survey-display-485.cfm

11. World Health Organization, UNICEF, United Nations, Department of Economic and Social Affairs, Population Division, World Bank. Trends in maternal mortality: 1990 to 2015 : estimates by WHO, UNICEF, UNFPA, World Bank Group and the United Nations Population Division. 2015. Available: http://www.who.int/reproductivehealth/publications/monitoring/maternal-mortality-2015/en/

12. MINISTRY OF HEALTH AND SOCIAL WELFARE. United Republic of Tanzania Ministry of Health and Social Welfare, National Family Planning Costed Implementation Program, 2010– 2015, Dar es Salaam, Tanzania: Reproductive and Child Health Section, 2010. 2010.

13. UNICEF. Maternal and child health fact sheet. Nov 2017 [cited 2 Jan 2020]. Available: https://www.unicef.org/tanzania/reports/maternal-and-child-health-fact-sheet

14. Tafere TE, Afework MF, Yalew AW. Counseling on family planning during ANC service increases the likelihood of postpartum family planning use in Bahir Dar City Administration, Northwest Ethiopia: a prospective follow up study. Contraception and Reproductive Medicine. 2018;3: 28. doi:10.1186/s40834-018-0081-x

15. FP 2020. Tanzania | Family Planning 2020 Commitment. 2013 [cited 8 Oct 2019]. Available: https://www.familyplanning2020.org/tanzania

16. Dynes MM, Bernstein E, Morof D, Kelly L, Ruiz A, Mongo W, et al. Client and provider factors associated with integration of family planning services among maternal and reproductive health clients in Kigoma Region, Tanzania: a cross-sectional study, April–July 2016. Reproductive Health. 2018;15: 152. doi:10.1186/s12978-018-0593-5

17. Kamhawi S, Underwood C, Murad H, Jabre B. Client-centered counseling improves client satisfaction with family planning visits: evidence from Irbid, Jordan. Glob Health Sci Pract. 2013;1: 180–192. doi:10.9745/GHSP-D-12-00051

18. Obare F, Liambila W, Birungi H. Family planning counselling and use among clients seeking abortion services in private health facilities in Kenya. 2014;28: 12.

19. Kakoko DC, Ketting E, Kamazima SR, Ruben R. Provision of Family Planning Services in Tanzania: A Comparative Analysis of Public and Private Facilities. African Journal of Reproductive Health / La Revue Africaine de la Santé Reproductive. 2012;16: 140–148.

20. Elewonibi B, Sato R, Manongi R, Msuya S, Shah I, Canning D. The distance-quality trade-off in women's choice of family planning provider in North Eastern Tanzania. BMJ Global Health. 2020;5: e002149. doi:10.1136/bmjgh-2019-002149

21. Nagai M, Bellizzi S, Murray J, Kitong J, Cabral EI, Sobel HL. Opportunities lost: Barriers to increasing the use of effective contraception in the Philippines. PLOS ONE. 2019;14: e0218187. doi:10.1371/journal.pone.0218187

22. Shu D, Young JG, Toh S. Privacy-protecting estimation of adjusted risk ratios using modified Poisson regression in multi-center studies. BMC Med Res Methodol. 2019;19: 228. doi:10.1186/s12874-019-0878-6

23. Zou G. A Modified Poisson Regression Approach to Prospective Studies with Binary Data. Am J Epidemiol. 2004;159: 702–706. doi:10.1093/aje/kwh090

24. Puri MC, Moroni M, Pearson E, Pradhan E, Shah IH. Investigating the quality of family planning counselling as part of routine antenatal care and its effect on intended postpartum contraceptive method choice among women in Nepal. BMC Women's Health. 2020;20: 29. doi:10.1186/s12905-020-00904-y

25. Kanyangarara M, Sakyi K, Laar A. Availability of integrated family planning services in HIV care and support sites in sub-Saharan Africa: a secondary analysis of national health facility surveys. Reproductive Health. 2019;16: 60. doi:10.1186/s12978-019-0713-x

26. TDHS. Tanzania Demographic and Health Survey and Malaria Indicator Survey 2015-2016. National Bureau of Statistics, Dar es Salaam, Tanzania and Rockville, Maryland, USA: MoHCDGEC, MoH, NBS, OCGS, and ICF; 2015 2016. Available: https://www.dhsprogram.com/pubs/pdf/SR233/SR233.pdf

27. Tafere TE, Afework MF, Yalew AW. Counseling on family planning during ANC service increases the likelihood of postpartum family planning use in Bahir Dar City Administration, Northwest Ethiopia: a prospective follow up study. Contracept Reprod Med. 2018;3: 1–9. doi:10.1186/s40834-018-0081-x

28. Achwoka D, Pintye J, McGrath CJ, Kinuthia J, Unger JA, Obudho N, et al. Uptake and correlates of contraception among postpartum women in Kenya: results from a national cross-sectional survey. Contraception. 2018;97: 227–235. doi:10.1016/j.contraception.2017.10.001

29. Do M, Kurimoto N. Women's Empowerment and Choice of Contraceptive Methods in Selected African Countries. IPSRH. 2012;38: 023–033. doi:10.1363/3802312

30. Dehingia N, Dixit A, Averbach S, Choudhry V, Dey A, Chandurkar D, et al. Family planning counseling and its associations with modern contraceptive use, initiation, and continuation in rural Uttar Pradesh, India. Reproductive Health. 2019;16: 178. doi:10.1186/s12978-019-0844-0

31. Maonga AR, Mahande MJ, Damian DJ, Msuya SE. Factors Affecting Exclusive Breastfeeding among Women in Muheza District Tanga Northeastern Tanzania: A Mixed Method Community Based Study. Matern Child Health J. 2016;20: 77–87. doi:10.1007/s10995-015-1805-z

32. Tumlinson K, Okigbo CC, Speizer IS. Provider barriers to family planning access in urban Kenya. Contraception. 2015;92: 143–151. doi:10.1016/j.contraception.2015.04.002

33. Clements S, Madise N. Who Is Being Served Least by Family Planning Providers? A Study of Modern Contraceptive Use in Ghana, Tanzania and Zimbabwe. African Journal of Reproductive Health / La Revue Africaine de la Santé Reproductive. 2004;8: 124–136. doi:10.2307/3583186

Point by point response to reviewer's comments

Manuscript ID: Ms. [**PONE-D-20-36870**]

Missed opportunity for family planning counselling along the continuum of care in Arusha region, Tanzania

Journal: PLOS ONE

Journal Requirements:

When submitting your revision, we need you to address these additional requirements.

1. Please ensure that your manuscript meets PLOS ONE's style requirements, including those for file naming. The PLOS ONE style templates can be found at

https://journals.plos.org/plosone/s/file?id=wjVg/PLOSOne_formatting_sample_main_body.pdf
and
https://journals.plos.org/plosone/s/file?id=ba62/PLOSOne_formatting_sample_title_authors_affiliations.pdf

Thanks for this important observation. We have now made the necessary changes in the revised manuscript.

2. Thank you for stating in your financial disclosure:

"This project was made possible by a grant from an anonymous donor to Harvard T.H. Chan School of Public Health. The funder had no role in study design, data collection and analysis, decision to publish, or preparation of the manuscript."
PLOS ONE requires you to include in your manuscript further information about the funder so that any relevant competing interests can be assessed. Please respond to the following questions:

1. Please state whether any of the research costs or authors' salaries were funded, in whole or in part, by a tobacco company (our policy on tobacco funding is at http://journals.plos.org/plosone/s/disclosure-of-funding-sources)
2. Please state whether the donor has any competing interests in relation to this work (see http://journals.plos.org/plosone/s/competing-interests) .
3. Please state whether the identity of the donor might be considered relevant to editors or reviewers' assessment of the validity of the work.
4. If the donors have no perceived or actual competing interests, please state: "The authors are not aware of any competing interests".

This information should be included in your cover letter. We will amend your financial disclosure and competing interests on your behalf.

Thank you for the comment. None of the research costs or authors' salaries were funded, in whole or in part, by a tobacco company. The identity of the donor is irrelevant to editors or reviewers' assessment of the validity of the work. The authors are not aware of any competing interests. This additional information has been updated in the cover letter and the revised manuscript.

3. Please provide additional details regarding participant consent. In the ethics statement in the Methods and online submission information, please ensure that you have specified what type you obtained (for instance, written or verbal, and if verbal, how it was documented and witnessed). If your study included minors, state whether you obtained consent from parents or guardians. If the need for consent was waived by the ethics committee, please include this information.

Thank you for this important observation. A written participant consent was obtained before participation in the study for women aged 18 years and older. Participants who were aged 16 – 17 an assent was requested. Consent for them to participate was sought from partners for those who were married/ cohabiting, and from parents/ guardians for those who were under parental care. This information has been added to the methods section of the revised manuscript.

4. Please consider whether participants younger than 18 years old should be referred to as 'girls' rather than 'women'.

We thank the editor for this important observation. We have now addressed this in the revised manuscript.

5. We noticed you have some minor occurrence of overlapping text with the following previous publication(s), which needs to be addressed:

- Nabirye, J., Matovu, J.K.B., Bwanika, J.B. et al. Missed opportunities for family planning counselling among HIV-positive women receiving HIV Care in Uganda. BMC Women's Health 20, 91 (2020). https://doi.org/10.1186/s12905-020-00942-6

In your revision ensure you cite all your sources (including your own works), and quote or rephrase any duplicated text outside the methods section. Further consideration is dependent on these concerns being addressed.

The authors thank the editor for this observation. We have now made the necessary citations in the revised manuscript.

Comments to the Author

1. Is the manuscript technically sound, and do the data support the conclusions?
The manuscript must describe a technically sound piece of scientific research with data that supports the conclusions. Experiments must have been conducted rigorously, with appropriate controls, replication, and sample sizes. The conclusions must be drawn appropriately based on the data presented.

Reviewer #1: Partly
Reviewer #2: Yes

---

2. Has the statistical analysis been performed appropriately and rigorously?

Reviewer #1: Yes

Reviewer #2: Yes

---

3. Have the authors made all data underlying the findings in their manuscript fully available?

The PLOS Data policy requires authors to make all data underlying the findings described in their manuscript fully available without restriction, with rare exception (please refer to the Data Availability Statement in the manuscript PDF file). The data should be provided as part of the manuscript or its supporting information, or deposited to a public repository. For example, in addition to summary statistics, the data points behind means, medians and variance measures should be available. If there are restrictions on publicly sharing data—e.g. participant privacy or use of data from a third party—those must be specified.

Reviewer #1: Yes

Reviewer #2: No

---

4. Is the manuscript presented in an intelligible fashion and written in standard English?

PLOS ONE does not copyedit accepted manuscripts, so the language in submitted articles must be clear, correct, and unambiguous. Any typographical or grammatical errors should be corrected at revision, so please note any specific errors here.

Reviewer #1: Yes
Reviewer #2: Yes

---

5. Review Comments to the Author

Please use the space provided to explain your answers to the questions above. You may also include additional comments for the author, including concerns about dual publication, research ethics, or publication ethics. (Please upload your review as an attachment if it exceeds 20,000 characters)

Reviewer #1: Missed opportunity for family planning counselling along the continuum of care in Arusha region, Tanzania.

Abstract

L 6&7: Associated factors of what: behavioral of the women or the health provider? What about health worker knowledge and skills, commodity supply, distance to the health facility, please clarify

Please define women of reproductive age (15-49 years of age).

We thank the reviewer for the observation, we have made the additions and defined the women of reproductive age. The authors acknowledge that apart from the women's behavioral factors studied in this paper, there could be other factors that could contribute. However, the Health worker and/or health facility related factors were not observed or studied at this time in this study project.

Methods

L 11: I suggest 'face-to-face'

We thank the reviewer for the comment, we have made the revision

L 11: Continue with to the abbreviation 'FP' after it has been introduced

We thank the reviewer for the comment, we have made the revision

L 13: The FP counselling: is it a group or one-on-one (confidential) counselling during the clinic visit?

We thank the reviewer for this important comment. FP counselling is done on a one-on-one basis between health care worker and client. Although in some instances general information on FP can be provided as group, but later on detailed information and client is given time to ask any questions or concerns.

L 16: Would contraceptive prevalence rate (CPR) or even modern CPR (mCPR) be more appropriate?

We thank the reviewer for this comment. Contraceptive Prevalence Rate (CPR) or modern CPR (mCPR) will tell us the extent or current usage of contraceptive or modern contraceptive. To determine the association between contraceptive usage and receipt of FP counselling, we implored a modified poisson regression model, whereas Prevalence Ratio (PR) was estimated.

Results

L 18: The percentage is sometime cited as '38%' and sometime as "40%" Please clarify

We thank the reviewer for the comment, we have made the revision

Conclusion

L 29: What about the other associated factors that is mentioned in the introduction. Were there any outcomes?

We thank the reviewer for the comment, we have made the revision

Introduction
L 12-19: I suggest taking above before the background reference status of FP situation in TZ
We thank the reviewer for the comment, we have made the changes
Page 4, L 13: the study also assesse'd' whether…
We thank the reviewer for the comment, we have made the revision

Materials and methods
L 16: Please stay with the abbreviation WRA
We thank the reviewer for the comment, we have made the editions in the revised manuscript
L 19: reference the data source: e.g. ministry of health or other partners
We thank the reviewer for the comment, we have added the reference
L 21: I suggest to delete "the"
We have made the deletion, thanks for the observation
Page 5, L 15: Are these referring to the "other associated factors"?
Thanks for the comment. Other associated factors include the demographics and other behavioral characteristics of the women.
L 16, L17 and L19: keep to the abbreviation introduced "FP"
We thank the reviewer for the comment, we have made the revisions
L 21: the outcome of L18-21 did not also come out in the conclusion
Thanks for the comment. The secondary outcome contraceptive usage was used to determine the association between receipt of FP counselling and current contraceptive usage. We found a significant association between receipt of FP counselling at any PNC visit and current usage of contraception (see Table 3). Conclusion was revised.
L 22: I suggest to include 'as shown in fig 1'.
We thank the reviewer for the important observation. We have placed the figure in the data collection section, where sample selection was described.

Sample selection description flow chart
L 1: FP counselling of (1,256/3,116) is 40% not 38%.
We thank the reviewer for the comment, we have made the revisions
L7-10, please use a space before the opening bracket
If the analysis is done up this category, the coverage should be 40% instead of 38%.
We thank the reviewer for the comment, we have made the revisions

Characteristic of the study participants
L 7: This is confusing, if 462 was excluded then, does it mean analysis for coverage was done on "2,654" out of the 3,116, then coverage of 38% is still questionable.
We thank the reviewer for the comment. The 462 women who were never pregnant in the past two years, were excluded in the separate analysis that looked on predictors of FP counselling at any ANC and PNC visits. The revisions are made in the revised manuscript.

Table 1: Characteristic of the study participants (N=3,116)
This should be discussed as one of the associated factors to FP counselling coverage in the conclusion
We thank the reviewer for the comment, we have made the revisions
Please double check your subtotals, for example age=3115, marital status=3,115, work/employed

only = 3114, etc
Thanks for the comment, we have double checked the subtotals. Age and marital status had one missing value in these variables, where employment status had two missing values.

Predictor of receipt of family planning counselling along the continuum of care
L 2: keep to the abbreviation "FP"
We thank the reviewer for the comment, we have made the revision
L 3: it is correctly stated here but in the Abstract, it is stated as 38%. Please don't use the word 'about' so the sentence needs reframing.
We thank the reviewer for the comment, we have made the revision

Discussion
L 4: Staying with the abbreviation WRA
We have made the revision, thanks for the comment.
L 7: Please discuss further, as table 1 show combined % for poorest and poorer as 24.2%, does this mean low FP counselling coverage is found among the middle to richest category; this also goes for employment.
We thank the reviewer for the comment, we have revised the discussion to include detailed information in the revised manuscript.
L 10: here again the 38% instead of 40%
We thank the reviewer for the comment, we have made the revision

Strength and Limitations
L 5: If these factors were not assessed, then why are they mentioned in the abstract "aim" to identify the associated factors.
Thanks for the comment. The study assessed the predictors of receiving FP counselling and determined to see if there is any association between receiving of FP counselling and Current contraceptive usage. However, the study did not look at the reasons why some women did not use contraception even after receiving FP counselling.
Conclusion
L 11: Here again the coverage is not consistent in the other section such as the conclusion in the abstract.
We thank the reviewer for the comment, we have made the revision

Reviewer #2:
The title refers to continuum of care while only ANC and postpartum periods are considered!
Thanks for the comment. The study assessed FP counselling at any health facility visit, at any ANC visit and at any PNC visit in the past two years.

It is not clear how 38% was calculated since 3115 women were included in the analysis "Overall, 1,256 (38%) women reported that they received FP counseling at any of the health facility visits in the past two years.
We thank the reviewer for the comment, we have made the revision in the revised manuscript.

Of the 1,389 and 1,409 women who had contact with the service delivery points for ANC and

PNC visits respectively; 27% and 26% had a missed FP counseling at ANC and PNC visit respectively." Calculations of 27 and 26% are also not clear!
We thank the reviewer for the comment, we have made the revision in the revised manuscript after double checking the calculations.

"The data were originally collected for an impact evaluation of a community-based intervention on contraceptive information, counselling and referral." Are the results presented in this paper before the intervention or after the intervention?
Thanks for the question. The results presented in the paper were for the baseline data before the intervention.

"A total of 3,116 women were included in the overall analysis; whereas 462 women who had no pregnancy were excluded for the ANC and PNC analysis." In figure 1, WRA included in the analysis was 3115, where did the extra participant come from to make them 3116?
Thanks for the comment. Figure one shows a total of 3,116 WRA included in the analysis (see Fig 1)

Where did the 462 women excluded come from since in figure 1, they are not shown and pregnancy was not mentioned as an exclusion criteria?
We thank the reviewer for the comment. The 462 women who were never pregnant in the past two years, were excluded in a separate sub-analysis that looked on predictors of FP counselling at any ANC and PNC visits. The clarifications are made in the revised manuscript.

"Overall, among the women who had had births in the last 30 months more than seventy percent received FP counselling at ANC and PNC visits (73% and 74% respectively)." The reported percentage in the results section are different from those in the abstract, since it is reported that only 38% received FP counselling.
We thank the reviewer for this important observation. Among the women that visited the health facility for any health-related visit in the past two years, 40% received FP counselling. Among the women who had had births in the last 30 months; 1,389 and 1,409 women had contact with the service delivery points for ANC and PNC visits respectively. Of these 68.8% and 73.6% received FP counselling at ANC and PNC visits respectively. Necessary revisions were made in the revised manuscript.

In the discussion, it is not clear what the authors are saying in this statement "These findings imply that 61% of the women missed FP counselling when they visited the facility for any health reason; while 26.8% and 26.2% missed FP counselling at 1 ANC and post-natal care (PNC) visits respectively." The discussion needs to be redone once the results have been revised.
We thank the reviewer for this important observation. Among the women that visited the health facility for any health-related visit in the past two years, only 40% received FP counselling. This means that 60% of the women that visited the health facility for any health-related visit did not get FP counselling. Among the women who had had births in the last 30 months; 1,389 and 1,409 women had contact with the service delivery points for ANC and PNC visits respectively. Of these 31.2% and 26.4% missed FP counselling at ANC and PNC visits respectively. Necessary revisions were made in the revised manuscript.
