# Supplementary material for: Missed opportunity for family planning counselling along the continuum of care in Arusha region, Tanzania
Source: PLoS One. 2021 Jul 14;16(7):e0250988. doi: 10.1371/journal.pone.0250988 (PMC8279341; doi:10.1371/journal.pone.0250988)
Supplement: Attachment — Submitted filename: Response to Reviewers.docx [file pone.0250988.s002.docx]

**Point by point response to reviewer’s comments**

**Manuscript ID:** Ms.  [**PONE-D-20-36870**]

**Missed opportunity for family planning counselling along the continuum of care in Arusha region, Tanzania**

**Journal:** PLOS ONE

**Journal Requirements:**

 When submitting your revision, we need you to address these additional requirements.

1. Please ensure that your manuscript meets PLOS ONE's style requirements, including those for file naming. The PLOS ONE style templates can be found at

<https://journals.plos.org/plosone/s/file?id=wjVg/PLOSOne_formatting_sample_main_body.pdf> and <https://journals.plos.org/plosone/s/file?id=ba62/PLOSOne_formatting_sample_title_authors_affiliations.pdf>

Thanks for this important observation. We have now made the necessary changes in the revised manuscript.

2. Thank you for stating in your financial disclosure:

"This project was made possible by a grant from an anonymous donor to Harvard T.H. Chan School of Public Health. The funder had no role in study design, data collection and analysis, decision to publish, or preparation of the manuscript."

PLOS ONE requires you to include in your manuscript further information about the funder so that any relevant competing interests can be assessed. Please respond to the following questions:

1. Please state whether any of the research costs or authors' salaries were funded, in whole or in part, by a tobacco company (our policy on tobacco funding is at http://journals.plos.org/plosone/s/disclosure-of-funding-sources)
2. Please state whether the donor has any competing interests in relation to this work (see http://journals.plos.org/plosone/s/competing-interests) .
3. Please state whether the identity of the donor might be considered relevant to editors or reviewers’ assessment of the validity of the work.
4. If the donors have no perceived or actual competing interests, please state: “The authors are not aware of any competing interests”.

This information should be included in your cover letter. We will amend your financial disclosure and competing interests on your behalf.

Thank you for the comment. None of the research costs or authors' salaries were funded, in whole or in part, by a tobacco company. The identity of the donor is irrelevant to editors or reviewers’ assessment of the validity of the work. The authors are not aware of any competing interests. This additional information has been updated in the cover letter and the revised manuscript.

3. Please provide additional details regarding participant consent. In the ethics statement in the Methods and online submission information, please ensure that you have specified what type you obtained (for instance, written or verbal, and if verbal, how it was documented and witnessed). If your study included minors, state whether you obtained consent from parents or guardians. If the need for consent was waived by the ethics committee, please include this information.

Thank you for this important observation. A written participant consent was obtained before participation in the study for women aged 18 years and older. Participants who were aged 16 – 17 an assent was requested. Consent for them to participate was sought from partners for those who were married/ cohabiting, and from parents/ guardians for those who were under parental care. This information has been added to the methods section of the revised manuscript.

4. Please consider whether participants younger than 18 years old should be referred to as 'girls' rather than 'women'.

 We thank the editor for this important observation. We have now addressed this in the revised manuscript.

5. We noticed you have some minor occurrence of overlapping text with the following previous publication(s), which needs to be addressed:

- Nabirye, J., Matovu, J.K.B., Bwanika, J.B. et al. Missed opportunities for family planning counselling among HIV-positive women receiving HIV Care in Uganda. BMC Women's Health 20, 91 (2020). <https://doi.org/10.1186/s12905-020-00942-6>

In your revision ensure you cite all your sources (including your own works), and quote or rephrase any duplicated text outside the methods section. Further consideration is dependent on these concerns being addressed.

 The authors thank the editor for this observation. We have now made the necessary citations in the revised manuscript.

**Comments to the Author**

1. Is the manuscript technically sound, and do the data support the conclusions?
The manuscript must describe a technically sound piece of scientific research with data that supports the conclusions. Experiments must have been conducted rigorously, with appropriate controls, replication, and sample sizes. The conclusions must be drawn appropriately based on the data presented.

Reviewer #1: Partly

Reviewer #2: Yes

 2. Has the statistical analysis been performed appropriately and rigorously?

 Reviewer #1: Yes

Reviewer #2: Yes

 3. Have the authors made all data underlying the findings in their manuscript fully available?

The [PLOS Data policy](http://www.plosone.org/static/policies.action#sharing) requires authors to make all data underlying the findings described in their manuscript fully available without restriction, with rare exception (please refer to the Data Availability Statement in the manuscript PDF file). The data should be provided as part of the manuscript or its supporting information, or deposited to a public repository. For example, in addition to summary statistics, the data points behind means, medians and variance measures should be available. If there are restrictions on publicly sharing data—e.g. participant privacy or use of data from a third party—those must be specified.

 Reviewer #1: Yes

Reviewer #2: No

 4. Is the manuscript presented in an intelligible fashion and written in standard English?

PLOS ONE does not copyedit accepted manuscripts, so the language in submitted articles must be clear, correct, and unambiguous. Any typographical or grammatical errors should be corrected at revision, so please note any specific errors here.

 Reviewer #1: Yes

Reviewer #2: Yes

**5. Review Comments to the Author**

Please use the space provided to explain your answers to the questions above. You may also include additional comments for the author, including concerns about dual publication, research ethics, or publication ethics. (Please upload your review as an attachment if it exceeds 20,000 characters)

**Reviewer #1: Missed opportunity for family planning counselling along the continuum of care in Arusha region, Tanzania.**
Abstract
L 6&7: Associated factors of what: behavioral of the women or the health provider? What about health worker knowledge and skills, commodity supply, distance to the health facility, please clarify
Please define women of reproductive age (15-49 years of age).
We thank the reviewer for the observation, we have made the additions and defined the women of reproductive age. The authors acknowledge that apart from the women’s behavioral factors studied in this paper, there could be other factors that could contribute. However, the Health worker and/or health facility related factors were not observed or studied at this time in this study project.

Methods
L 11: I suggest ‘face-to-face’

We thank the reviewer for the comment, we have made the revision

L 11: Continue with to the abbreviation ‘FP’ after it has been introduced

We thank the reviewer for the comment, we have made the revision

L 13: The FP counselling: is it a group or one-on-one (confidential) counselling during the clinic visit?

We thank the reviewer for this important comment. FP counselling is done on a one-on-one basis between health care worker and client. Although in some instances general information on FP can be provided as group, but later on detailed information and client is given time to ask any questions or concerns.

L 16: Would contraceptive prevalence rate (CPR) or even modern CPR (mCPR) be more appropriate?
We thank the reviewer for this comment. Contraceptive Prevalence Rate (CPR) or modern CPR (mCPR) will tell us the extent or current usage of contraceptive or modern contraceptive. To determine the association between contraceptive usage and receipt of FP counselling, we implored a modified poisson regression model, whereas Prevalence Ratio (PR) was estimated.

Results
L 18: The percentage is sometime cited as ‘38%’ and sometime as "40%" Please clarify
We thank the reviewer for the comment, we have made the revision
Conclusion
L 29: What about the other associated factors that is mentioned in the introduction. Were there any outcomes?
We thank the reviewer for the comment, we have made the revision

Introduction
L 12-19: I suggest taking above before the background reference status of FP situation in TZ

We thank the reviewer for the comment, we have made the changes
Page 4, L 13: the study also assesse’d’ whether…
We thank the reviewer for the comment, we have made the revision

Materials and methods
L 16: Please stay with the abbreviation WRA

We thank the reviewer for the comment, we have made the editions in the revised manuscript
L 19: reference the data source: e.g. ministry of health or other partners

We thank the reviewer for the comment, we have added the reference
L 21: I suggest to delete "the"
We have made the deletion, thanks for the observation

Page 5, L 15: Are these referring to the "other associated factors"?

Thanks for the comment. Other associated factors include the demographics and other behavioral characteristics of the women.

L 16, L17 and L19: keep to the abbreviation introduced "FP"

We thank the reviewer for the comment, we have made the revisions
L 21: the outcome of L18-21 did not also come out in the conclusion

Thanks for the comment. The secondary outcome contraceptive usage was used to determine the association between receipt of FP counselling and current contraceptive usage. We found a significant association between receipt of FP counselling at any PNC visit and current usage of contraception (see Table 3). Conclusion was revised.

L 22: I suggest to include ‘as shown in fig 1’.
We thank the reviewer for the important observation. We have placed the figure in the data collection section, where sample selection was described.

Sample selection description flow chart
L 1: FP counselling of (1,256/3,116) is 40% not 38%.

We thank the reviewer for the comment, we have made the revisions
L7-10, please use a space before the opening bracket
If the analysis is done up this category, the coverage should be 40% instead of 38%.
We thank the reviewer for the comment, we have made the revisions

Characteristic of the study participants
L 7: This is confusing, if 462 was excluded then, does it mean analysis for coverage was done on "2,654" out of the 3,116, then coverage of 38% is still questionable.
We thank the reviewer for the comment. The 462 women who were never pregnant in the past two years, were excluded in the separate analysis that looked on predictors of FP counselling at any ANC and PNC visits. The revisions are made in the revised manuscript.

Table 1: Characteristic of the study participants (N=3,116)
This should be discussed as one of the associated factors to FP counselling coverage in the conclusion

We thank the reviewer for the comment, we have made the revisions
Please double check your subtotals, for example age=3115, marital status=3,115, work/employed only = 3114, etc
Thanks for the comment, we have double checked the subtotals. Age and marital status had one missing value in these variables, where employment status had two missing values.

Predictor of receipt of family planning counselling along the continuum of care
L 2: keep to the abbreviation "FP"

We thank the reviewer for the comment, we have made the revision
L 3: it is correctly stated here but in the Abstract, it is stated as 38%. Please don’t use the word ‘about’ so the sentence needs reframing.
We thank the reviewer for the comment, we have made the revision

Discussion
L 4: Staying with the abbreviation WRA

We have made the revision, thanks for the comment.
L 7: Please discuss further, as table 1 show combined % for poorest and poorer as 24.2%, does this mean low FP counselling coverage is found among the middle to richest category; this also goes for employment.

We thank the reviewer for the comment, we have revised the discussion to include detailed information in the revised manuscript.
L 10: here again the 38% instead of 40%
We thank the reviewer for the comment, we have made the revision

Strength and Limitations
L 5: If these factors were not assessed, then why are they mentioned in the abstract "aim" to identify the associated factors.
Thanks for the comment. The study assessed the predictors of receiving FP counselling and determined to see if there is any association between receiving of FP counselling and Current contraceptive usage. However, the study did not look at the reasons why some women did not use contraception even after receiving FP counselling.
Conclusion
L 11: Here again the coverage is not consistent in the other section such as the conclusion in the abstract.

We thank the reviewer for the comment, we have made the revision

**Reviewer #2:**

The title refers to continuum of care while only ANC and postpartum periods are considered! Thanks for the comment. The study assessed FP counselling at any health facility visit, at any ANC visit and at any PNC visit in the past two years.

It is not clear how 38% was calculated since 3115 women were included in the analysis "Overall, 1,256 (38%) women reported that they received FP counseling at any of the health facility visits in the past two years.

We thank the reviewer for the comment, we have made the revision in the revised manuscript.

Of the 1,389 and 1,409 women who had contact with the service delivery points for ANC and PNC visits respectively; 27% and 26% had a missed FP counseling at ANC and PNC visit respectively." Calculations of 27 and 26% are also not clear!

We thank the reviewer for the comment, we have made the revision in the revised manuscript after double checking the calculations.

"The data were originally collected for an impact evaluation of a community-based intervention on contraceptive information, counselling and referral." Are the results presented in this paper before the intervention or after the intervention?

Thanks for the question. The results presented in the paper were for the baseline data before the intervention.

"A total of 3,116 women were included in the overall analysis; whereas 462 women who had no pregnancy were excluded for the ANC and PNC analysis." In figure 1, WRA included in the analysis was 3115, where did the extra participant come from to make them 3116?

Thanks for the comment. Figure one shows a total of 3,116 WRA included in the analysis (see Fig 1)

Where did the 462 women excluded come from since in figure 1, they are not shown and pregnancy was not mentioned as an exclusion criteria?

We thank the reviewer for the comment. The 462 women who were never pregnant in the past two years, were excluded in a separate sub-analysis that looked on predictors of FP counselling at any ANC and PNC visits. The clarifications are made in the revised manuscript.

"Overall, among the women who had had births in the last 30 months more than seventy percent received FP counselling at ANC and PNC visits (73% and 74% respectively)." The reported percentage in the results section are different from those in the abstract, since it is reported that only 38% received FP counselling.

We thank the reviewer for this important observation. Among the women that visited the health facility for any health-related visit in the past two years, 40% received FP counselling. Among the women who had had births in the last 30 months; 1,389 and 1,409 women had contact with the service delivery points for ANC and PNC visits respectively. Of these 68.8% and 73.6% received FP counselling at ANC and PNC visits respectively. Necessary revisions were made in the revised manuscript.

In the discussion, it is not clear what the authors are saying in this statement "These findings imply that 61% of the women missed FP counselling when they visited the facility for any health reason; while 26.8% and 26.2% missed FP counselling at 1 ANC and post-natal care (PNC) visits respectively." The discussion needs to be redone once the results have been revised.

We thank the reviewer for this important observation. Among the women that visited the health facility for any health-related visit in the past two years, only 40% received FP counselling. This means that 60% of the women that visited the health facility for any health-related visit did not get FP counselling. Among the women who had had births in the last 30 months; 1,389 and 1,409 women had contact with the service delivery points for ANC and PNC visits respectively. Of these 31.2% and 26.4% missed FP counselling at ANC and PNC visits respectively. Necessary revisions were made in the revised manuscript.
